# Cortical dynamics are differentially associated with decision-making strategies in human wayfinding: An EEG study

**DOI:** 10.3758/s13415-025-01357-2

**Published:** 2025-11-06

**Authors:** Ju-Yi Huang, Otmar Bock, Clément Naveilhan, Stephen Ramanoël, Ronja V. Faßbender, Daniel Memmert, Oezguer A. Onur

**Affiliations:** 1 Institute of Exercise Training and Sport Informatics, German Sport University Cologne, Am Sportpark Müngersdorf 6, 50933 Köln Cologne, Germany; 2 LAMHESS, Université Côte d’Azur, Nice, France; 3 Department of Neurology, Faculty of Medicine and University Hospital Cologne, University of Cologne, Cologne, Germany

**Keywords:** EEG, Decision-making, Cognitive strategy, Spatial navigation, Wayfinding

## Abstract

Navigating urban environments involves making directional decisions at intersections, which can be guided by different cognitive strategies. This study investigated whether distinct strategies correspond to specific spatiotemporal brain activity patterns, reflecting differences in cognitive processes using source-localized scalp electroencephalography (EEG). Thirty-two participants navigated five strategy-specific virtual mazes and made directional decisions at intersections in each given maze. At the behavioral level, we found that strategies involving higher cognitive memory load were associated with lower accuracy, while those requiring complex visual processing resulted in longer reaction times. At the EEG level, we observed that increased theta activity was sensitive to differences in cognitive demands across strategies and was particularly associated with cue-piloting, memory retrieval, and spatial information updating and integration. Increased alpha activity was linked to visual cue and scene processing, while both increased and decreased beta activity reflected internally referenced memory and predictive processing, respectively. We further found that strategies relying on wisely visible cues showed similar spatiotemporal theta activation patterns, whereas those involving absent or nearby visual cues exhibited distinct patterns. Lastly, we observed that increased theta, alpha, and beta activity were associated with higher cognitive demand both before and after decision making. However, during decision making, increased theta activity was linked to efficient navigation in frontal-limbic-parietal-temporal regions and to greater cognitive demand in occipital regions. These findings indicate that cortical dynamics differ according to the cognitive strategy, depending on the type of visual cues and spatial judgments, thereby supporting a classification framework grounded in cognitive processing demands.

**Supplementary Information:**

The online version contains supplementary material available at 10.3758/s13415-025-01357-2.

## Introduction

Finding our way from a starting location to a target location is a fundamental aspect of everyday mobility and independence. This process can be challenging, as it involves the interplay of complex cognitive functions: we must integrate multimodal spatial information, store and recall spatial knowledge in multiple reference frames, and use this information to decide how to travel (for reviews, see Hegarty et al., 2023; Wolbers & Hegarty, 2010). In the present study, we focus on the dynamic neural correlates of the latter process, specifically the decision making at intersections encountered along a route.

Wayfinding decisions can be based on two types of cognitive strategies, typically classified according to how spatial information is encoded and utilized within egocentric or allocentric spatial reference frames (Hegarty et al., 2023). Response (egocentric) strategies rely on egocentric representation, in which information is encoded relative to a person’s own body position. Decisions are made by recalling previously learned associations between specific stimuli from the environment (e.g., turns or visual cues) and corresponding wayfinding responses along a route. Place (allocentric) strategies rely on allocentric representation, where spatial information is encoded independently of a person’s current position, often in the form of a mental map. These strategies involve understanding the spatial relationships between visual cues and their locations in the environment, allowing for taking shortcuts. However, although place strategies are inherently allocentric, active wayfinding requires continuously identifying one’s own location within the environment. This process involves the transformation of allocentric representations into egocentric perspectives to guide decision making (Ekstrom et al., 2017; Wolbers & Wiener, 2014).

The above classification of strategies based on spatial reference frames is strongly supported by neuroimaging studies (e.g., functional magnetic resonance imaging (fMRI)), which show that egocentric strategies are associated with parieto-striatal activity, whereas allocentric strategies rely on the hippocampus (see, e.g., Chersi & Burgess, 2015; Hegarty et al., 2023). However, recent behavioral and neural findings suggest that this dichotomy does not fully capture the complexity of human wayfinding (Chrastil et al., 2013). Instead, a more comprehensive understanding may lie in a detailed categorization of the cognitive subprocesses involved during wayfinding. These subprocesses reflect a dynamic interaction between general cognitive functions (e.g., attention, working memory, and sensory integration) and specific wayfinding processes, which vary according to task demands, available cues, and strategy use (Ekstrom et al., 2017; Hegarty et al., 2023). In the context of intersection decisions, these processes could be further broken down into a set of core cognitive subprocesses, each shown to correlate with distinct brain regions and related to specific EEG oscillations (see Table 1 for a detailed summary).

**Table 1:** Cognitive subprocesses and neural correlates underlying wayfinding decisions at intersections

Subprocess	Description	EEG correlate
**Approaching**		
Expectation formation	Anticipating what comes next based on prior spatial information and context	Midfrontal theta and alpha^1, 2^
Self-reference	Using own body position and perspective to interpret space	Frontal-parietal-premotor theta and alpha^3,4^
Scene processing and recognition	Perceiving visual features in contexts and interpreting those relevant features	Frontal-central and parietal-occipital theta and alpha^5, 6^
**Decision making**		
Memory retrieval	Retrieving stored spatial information relevant to make decision	Midfrontal theta; posterior parietal theta, alpha and beta^1, 7^
Spatial update and integration	Combining spatial cues to form a coherent mental representation of the environment	Frontal-parietal, retrosplenial cortex, medial parietal-temporal-occipital cortex theta and alpha^3, 4, 8, 9, 10, 11^
Response selection and motor execution	Decide directions and execute actions	Sensorimotor, supplementary motor cortex beta^3, 12^
**Feedback**		
Outcome monitoring and error detection	Monitoring and detecting errors or conflict between expected and actual outcomes	Frontal-midline theta^1, 13^
Feedback evaluation and reinforcement	Processing feedback and using as reward or error signals to adjust behavior	Frontal-midline and temporal theta^1, 13, 14^

*Note.* ^1^Chrastil et al. (2022); ^2^Du et al. (2023); ^3^Gramann et al. (2006); ^4^Lin et al., (2015); ^5^Cheng et al. (2022); ^6^Delaux et al. (2021); ^7^Jaiswal et al. (2011); ^8^Do et al. (2021); ^9^Gramann et al. (2021); ^10^Nguyen et al. (2014); ^11^White et al. (2012);^12^Tzagarakis et al. (2015); ^13^Lin et al. (2022); ^14^Cavanagh et al. (2010)

In our preceding study, we developed five strategy-specific mazes, each designed such that successful decision making was only possible using one specific cognitive strategy, not with the other four, at intersections (Bock et al., 2024; see the five mazes and their characteristics in Table 2). By correlating participants’ performance in different mazes, we found behavioral evidence for both strategy-specific and generalized wayfinding decision processes, but not for processes tied to a specific reference frame involved. In addition, our recent finding aligns with the study showing that the effects of age are strategy dependent rather than frame dependent. Specifically, we observed that strategies with higher cognitive demands are more negatively impacted by aging than those with lower demands (Huang et al., in press).

**Table 2:** Characteristics of five common cognitive strategies for wayfinding decisions at intersections

Strategy	Characteristic
**Rely on egocentric reference frame**	
Serial order strategy	Encoding the sequence of directions based on body turns
Associative cue strategy	Encoding the association between visual cues and turns
Beacon strategy	Searching a widely visible visual cue to make turn decisions
**Rely on egocentric and allocentric references frames**	
Relative location strategy	Searching widely visual cues and identifying a goal location based on their spatial relationship to make turn decisions
Cognitive map strategy	Encoding the mental representation of the spatial layout for wayfinding decisions

While previous research (see reviews by Boccia et al., 2014; Hegarty et al., 2023) has established that egocentric and allocentric cognitive strategies engage distinct brain regions, these findings have largely relied on neuroimaging methods with limited temporal resolution (e.g., fMRI), which constrain the ability to capture rapid neural dynamics during the wayfinding decision process. In addition, earlier studies used maze paradigms that allowed participants to either combine strategies or choose their preferred type (e.g., Dual Solution Paradigm: Marchette et al., 2011; Radial Arm Maze: Iaria et al., 2003). This flexibility makes it difficult to disentangle whether specific strategies, for example, serial order or associative cue strategy, rely on common or distinct cortical representations.

In the present study, we aimed to determine whether cognitive strategies are associated with distinct patterns of cortical activation during active wayfinding, regardless of whether they rely simply on an egocentric reference frame or require transformation from allocentric to egocentric perspectives. We hypothesized that each strategy, due to its unique cognitive demands, would elicit a different spatiotemporal pattern of brain activity. To test our hypothesis, we repurposed five virtual mazes developed for earlier wayfinding research (Bock et al., 2024) and recorded brain activity with electroencephalography (EEG). EEG offers high temporal resolution, enabling the capture of millisecond-level neural dynamics, which is more optimal for studying fast-paced cognitive processes during spatial decision making (see review by McLaren-Gradinaru et al., 2025). We focused on theta, alpha, and beta frequency bands and examined distinct temporal phases at intersections: approach, decision making, and feedback using the source localization method (see Table 1 for the associated cognitive subprocesses and corresponding cortical activation patterns for each phase).

## Method

### Participants

Thirty-two young adults (age range: 20–30 years, M = 25.1, SD = 2.6 years; 16 females; education: 14.7 ± 2.6 years) were recruited through distribution of flyers at local communities. All participants were right-handed and had no depression, alcohol abuse, or history of neurological disorders by self-report. Two participants were excluded from the EEG analysis due to technical issues resulting in incomplete EEG recordings. We calculated the required sample size using G*Power (Faul et al., 2007) and selected a medium effect size of 0.3, a significance level of α = 0.05, and a statistical power of 0.8, resulting in a required sample size of 15 participants. To account for the high variability in EEG data and ensure stable source reconstruction for cluster estimates, we decided to double the sample size to enhance the reliability and robustness of our findings. This study was part of a research program that was approved by the Ethics Commission of the German Sport University (approval no. Nr. 062/2020), and all procedures were carried out in compliance with the Declaration of Helsinki. All participants signed an informed consent statement before testing began.

### Wayfinding decision task and general procedure

Participants were asked to perform five wayfinding decision tasks while sitting comfortably in a dimly illuminated room in front of a 24-in. display monitor. Each task was implemented in a different, strategy-specific maze (details see below), constructed using Unreal Engine® (Epic Games, Inc.) and modified by Presentation® (Version 22.1, Neurobehavioral Systems, Inc., Berkeley, CA, USA) to display stimuli and record responses.

**Fig. 1:**
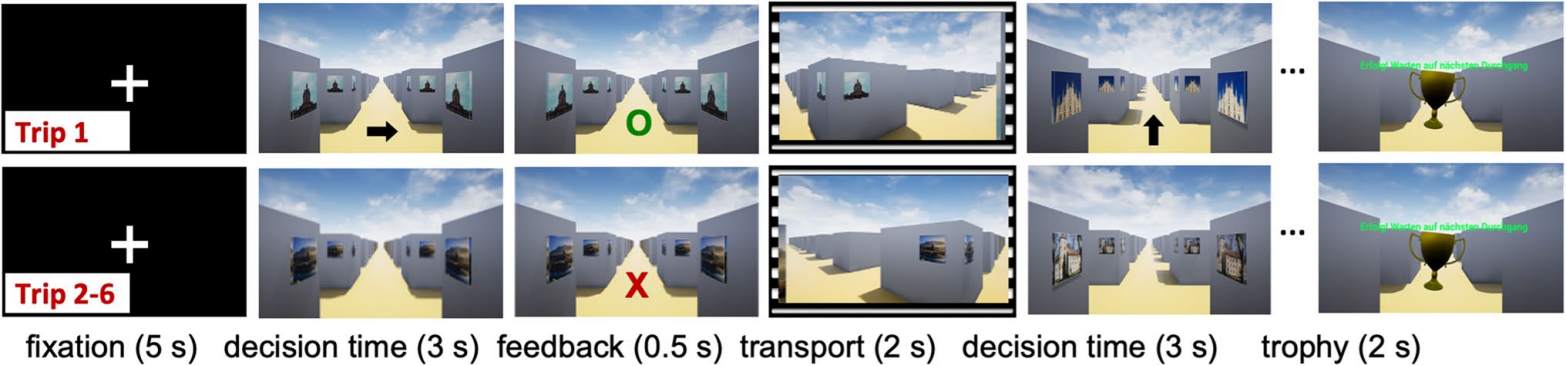
Exemplary snapshots of trip 1 (**top**) and trips 2–6 (**bottom**) from maze A. On trip 1, black arrows indicate the correct response. On all trips, feedback by green ‘o’ indicates a correct response, red ‘x’ an incorrect response, and red ‘?’ a missing response. Sprocket holes symbolize passive transport to the next intersection

Each participant took six trips through each maze. During the first trip, a guiding arrow was displayed on the maze floor throughout the decision time interval, indicating the direction in which the route continued (arrow-guided phase; see Fig. 1 top row, showing the arrow cue presented at decision time). During the subsequent five trips, no arrows were provided, and participants had to decide on their own which direction to take (self-guided phase; see Fig. 1 bottom row, showing the absence of an arrow cue at decision time). Each trip started with the display of a central cross for 5 s, followed by the display of an intersection for 3 s. During this latter interval, participants had to respond as quickly and accurately as possible by indicating the direction in which the route continued (i.e.*,* straight, left, or right) by tilting the joystick handle in the corresponding direction. They were instructed to only give one answer, anytime during the 3-s interval. After that interval, feedback was displayed for 0.5 s, indicating whether the response was correct, incorrect, or missing. Next, participants were passively transported at a constant speed in the correct direction, even if their response was incorrect or missing, until they stopped at the next intersection 2 s later. This intersection-feedback-transport cycle of events was repeated until the final intersection of the trip, where a trophy was displayed for 2 s as a virtual reward.

The next trip started automatically, after a 5-s break with a black screen. Once all six trips in a given maze were completed, participants closed their eyes and rested in a sitting position for 4 min. After that, the next maze was introduced, with a total of five mazes presented. To ensure balanced exposure, the order of the maze presentation was counterbalanced across participants using a Latin square design. An entire session lasted around 2.5–3 h, which included EEG preparation, task introduction, and about 90 min of testing with rest intervals.

### Strategy-specific mazes

**Fig. 2:**
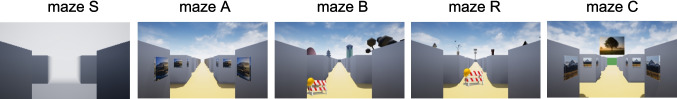
Exemplary snapshots of an intersection at decision-time interval in each maze. The external wall of maze C had a different color of each side (green, red, blue or purple, here green) to facilitate self-orientation

Each maze was tailored to enforce the usage of one specific decision-making strategy, as described by Bock et al. (2024). Figure 2 shows exemplary snapshots of an intersection in each maze.

In maze S (for Serial order strategy), participants were instructed to follow the same route across 12 intersections on each trip. All intersections looked the same, with no distinctive visual cues. Thus, to complete the task successfully, participants had to memorize the sequence of directions to take.

In maze A (for Associative cue strategy), participants were asked to proceed across 12 intersections, each characterized by a unique visual cue displayed at all corners. Each cue was associated with a particular direction to take, but the order of cues varied from trip to trip. Thus, to complete the task successfully, participants had to memorize the associations between cues and directions.

In maze B (for Beacon strategy), participants were instructed to approach the intersection located just in front of an exotic-looking tree, along the shortest possible route. The tree was one of 13 tall objects placed equidistantly around the maze, visible from anywhere within the maze. The starting position differed between trips, with the constraint that the shortest possible route always involved 12 decisions. Since many different shortest possible routes existed on each trip, one corridor of each intersection was blocked by a barrier to ensure that all participants took the same route, experienced the same visual stimulation, and produced comparable motor responses.

Maze R (for Relative location strategy) was similar to maze B, except that a different set of 13 tall objects was used, and the goal was defined as an imaginary point that formed an equilateral triangle with two of those objects (i.e.*,* tower and poles).

In maze C (for Cognitive map strategy), participants had to visit 12 intersections, each characterized by a unique visual cue displayed at all corners. Twelve intersections featuring visual cues alternated with intersections that provided no cues, with the total visited area forming a 5 × 7 grid. Once participants arrived at an intersection with a visual cue on the wall, the next-to-visit cue was shown floating above. The next-to-visit cue was always two intersections away, thus requiring two directional decisions: the first one to proceed left, right, or straight, and the second one to proceed straight on. The starting point of each trip was the same, but the order in which the cues had to be visited varied from trip to trip. Thus, memorizing the sequence of directions or cue-direction associations would be of no use; to complete the task successfully, participants therefore had to memorize the spatial layout of cues in the maze, and keep track of their own position and orientation with respect to the maze.

Performance on each trip was quantified as accuracy (i.e., the proportion of correct responses ranging from 0 = all incorrect to 1 = all correct). Reaction time for correct responses was measured as the interval between stopping at an intersection and moving the joystick.

### EEG data collection and pre-processing

We recorded continuous EEG signals from a total of 64 active AgCl electrodes, using an elastic cap (actiCAP SNAP) and a stationary actiCHamp EEG system (BrainProducts GmbH, Gilching, Germany), at a sampling rate of 1 kHz. The electrodes were positioned according to the international 10–20 system (Chatrian et al., 1985) and the average median electrode impedance across participants was 13.2 ± 6.4 kΩ, with an average of 6.05% of electrodes exceeding 25 kΩ (see Online Supplementary Material (OSM) Fig. 1 for impedance distributions). All electrodes were retained for further analysis, as the ideal target impedance is 25 kΩ and values up to 50 kΩ are acceptable for active wet electrodes (Brain Products GmbH, 2022; Laszlo et al., 2014; Mathewson et al., 2017). The ground electrode was placed at Fpz and one electrode was placed at FCz as a reference. Signals were recorded using BrainVision Recorder and event markers were sent and synchronized with EEG data stream from Presentation® software (Version 22.0, Neurobehavioral Systems, Inc., Berkeley, CA, USA) through the parallel port.

For data preprocessing, we exported data to MATLAB (R2024a; The MathWorks Inc., Natick, MA, USA) and conducted the standard processing using the EEGLAB toolbox (Delorme & Makeig, 2004) with customized *BeMoBIL* Pipeline scripts and functions (Klug et al., 2022). We first downsampled data to 256 Hz and removed non-maze-related data (i.e., resting periods and intervals between mazes). We then removed the line noise in data using the *Zapline-plus* plugin (Klug & Kloosterman, 2022), and noisy channels were identified and rejected using the *clean_rawdata* plugin, based on criteria of channel correlation with neighboring ones below 0.8 and more than 30% of the channel data flagged as bad, consistent with established recommendations in Bigdely-Shamlo et al. (2015). On average, 2.34 channels (SEM = 0.24) were rejected per participant. Rejected channels were reconstructed by spherical interpolation from neighboring channels and re-referenced to the common average. The above procedure reduced bias introduced by noisy data from bad channels and an unbalanced electrode montage when computing the common average reference, as recommended in established preprocessing pipelines (Bigdely-Shamlo et al., 2015). Subsequently, we applied a high-pass filter at 1.5 Hz to reduce slow drifts, following the recommendation for independent component analysis in Klug and Gramann (2021; see OSM: EEG Preprocessing Details (1) for filter specifications).

To clean the dataset, we used the adaptive mixture independent component analysis (AMICA) algorithm to decompose independent components (ICs), optimizing decomposition through 2,000 iterations with automatic bad sample rejection (Palmer et al., 2008). We then applied the ICLabel algorithm (Pion-Tonachini et al., 2019) to automatically classify sources as brain or non-brain activity and used the DIPFIT plugin to compute an equivalent current dipole model (ECD) to estimate the spatial origin of each IC (Oostenveld & Oostendorp, 2002). For this, we applied a common electrode location file and co-registered this file with a boundary element head model based on the MNI brain (Montreal Neurological Institute, Montreal, QC, Canada) to estimate dipole location. For each IC, the estimated dipole location serves as an approximation of its spatial origin. From the pool of ICs of each participant, we removed ICs related to ocular activity or muscle and extracted only those classified as brain activity with a probability ≥ 60%, balancing the inclusion of real neural signals while minimizing noise for further analysis (Naveilhan et al., 2025; Pion-Tonachini et al., 2019).

From the cleaned dataset, we first band-pass filtered the data with lower cut-off frequency of 0.25 Hz (with an order of 1,650 and a transition bandwidth of 0.5 Hz, leads a passband edge at 0.5 Hz) and an upper cut-off frequency of 40 Hz (with an order of 42 and transition bandwidth of 10 Hz, leads a passband edge at 30 Hz). Next, we epoched the data relative to stopping at intersections. Each epoch was extended, from −0.5 s before to 4 s after stops (i.e.*,* to 0.5 s after feedback), to capture the decision-making and memory processes central to the strategy employed in each maze. Epochs with an incorrect or missing response were excluded. Additionally, the *bemobil_reject_epochs* function (Klug et al., 2022) was employed to exclude the 5% most artifact-contaminated epochs for each participant. Finally, we performed time–frequency decomposition analysis with Superlets method (Moca et al., 2021) using FieldTrip’s *ft_freqanalysis* function, covering 2–46 Hz on a logarithmic scale with a width and Gaussian width of 2. This method improves the resolution of EEG data by combining multiple wavelets, enabling the detection of brief, event-related brain oscillations across a wide frequency range while preserving temporal precision. It captures transient neural events, such as theta bursts, that conventional spectral analyses often miss, overcoming the time–frequency trade-off inherent in classical methods like the short-time Fourier and traditional wavelet transforms (Bârzan et al., 2022; Moca et al., 2021).

### EEG analysis

#### Cluster identification

On average, each participant contributed 13 ICs. To balance over-segmentation, which can lead to undersampled clusters, and to maximize cluster separation, the optimal number of clusters (*k*) was evaluated over the range of 5–15 across 1,000 iterations using the Silhouette (Rousseeuw, 1987) and Davies-Bouldin (Davies & Bouldin, 1979) indices. These evaluations were based on the coordinates of equivalent current dipoles fitted to IC scalp topographies (i.e., weights in the columns of the mixing matrix). Both metrics consistently identified *k* = 15 as the most frequently optimal solution. Thus, ICs from all epochs were spatially clustered into 15 groups across participants and a 3 SD threshold was applied for outlier detection. The clustering solution was weighted based on features of the number of subjects (weight = 3), the number of ICs per subject (weight = −2), cluster spread (weight = −2), mean residual variance (RV) of ICs (weight = 2), and spread normality (weight = −1). To ensure reliability, we repeated the clustering process 5,000 times, ranked the solutions based on the combined metric score calculated from above weighted features, and selected the highest-ranking clustering result. From the selected solution, clusters containing ICs from fewer than 50% of participants were excluded to improve consistency in group-level statistical inference, even though this may omit meaningful but less common brain activity patterns (Bigdely-Shamlo et al., 2013; Huster et al., 2015). For clusters that contained more than one IC for a given participant, only the IC with the lowest RV was retained to ensure the most reliable representation of brain activity.

#### Time–frequency analysis

To compare brain activation patterns across mazes, we extracted single-trial time–frequency data for each IC within the frequency range of 0.5–30 Hz for each epoch, spanning from − 0.5 s to 4 s relative to the event. Given the continuous nature of the wayfinding task, which lacked a clear resting interval, we applied single-trial z-score normalization for baseline correction within each epoch (Grandchamp & Delorme, 2011). The normalized epochs were then averaged separately for each maze and cluster.

#### Mean power-activity analysis

To test our hypothesis, we calculated the mean power of theta (4–8 Hz), alpha (8–12 Hz), and beta (13–30 Hz) frequencies for each IC from the aforementioned time–frequency z-score data. This was done separately for three time blocks in relation to stop (0 s) at intersections: approach (−0.5 s to 0 s before stops), early decision (0 s to + 0.5 s, early stage after stops), and feedback (+ 3 s to + 3.5 s, end stage after stops). The later decision phase (+ 0.5 to + 3 s) was excluded to maintain temporal consistency across intersections, as it involves variable response times (see behavioral results) and encompasses overlapping processes such as movement execution (Makeig et al., 2004), post-decision processing (Philiastides & Sajda, 2006), and feedback anticipation (van de Vijver et al., 2011). These distinct neural processes generate divergent dynamics that may confound the interpretation of decision-related signals.

### Statistical analysis

#### Behavioral data analysis

For the behavioral data, we conducted two Friedman tests across mazes and calculated the effect size using Kendall’s W (W). The non-parametric Friedman tests were chosen since data distribution was not normal (Shapiro–Wilk tests indicated *p* <.05 for both variables). One test examined mean accuracy, and the other assessed mean reaction time, on self-guided trips. To pinpoint differences between specific mazes, we performed post hoc pairwise comparisons using Dunn’s tests. *P-*values were adjusted using the Bonferroni method to control for multiple comparisons and effect sizes were calculated using the rank-biserial correlation coefficient (*r*) for non-parametric data.

#### Time–frequency data analysis

To compare averaged time–frequency data across mazes, we performed permutation testing with 5,000 iterations (Maris & Oostenveld, 2007) on each data point (60 frequency points × 1,253 time windows), followed by post hoc pairwise comparisons between mazes. *P-*values of all outcomes were then corrected using the Threshold-Free Cluster Enhancement (TFCE) method. This method automatically identifies significant clusters of data, eliminating the need to set a fixed threshold for significance and controlling for potential errors due to multiple comparisons within the EEG data (Mensen & Khatami, 2013; Smith & Nichols, 2009). *P-*values were adjusted for multiple testing using the Bonferroni correction (*p* =.05/number of clusters, n = 13). Significance was reported for values below the threshold (*p* <.004) and effect sizes were reported using Cohen’s d (*d*).

#### Mean power activity data analysis

To address our main hypothesis, we conducted linear mixed-effects modeling on mean power activity across all mazes, as well as on subsets with pairwise maze comparisons, to identify spatiotemporal activation differences between mazes separately for each frequency band:theta, alpha, and beta. Each model included fixed effects for “maze” (S, A, B, R, C), “time phase” (approach, early decision, feedback), and “cluster” (cluster 1–13), along with their interactions, and a random intercept for subjects to account for within-subject variability. The model was specified as:

*mean power* ~ maze * time phase * cluster + *(1 | subject)*

Type III ANOVA statistics were then computed using Satterthwaite’s approximation from the model as implemented in the *lmerTest* package (Kuznetsova et al., 2017), and partial eta squared (η^2^_p_) values were calculated to estimate effect sizes using the *effectsize* package (Ben-Shachar et al., 2020). To control for multiple comparisons, *p*-values were adjusted using the Benjamini–Hochberg method.

#### Neural-behavioral correlation analysis

To link neural measurements with behavioral outcomes, we conducted correlation analyses between EEG power and two behavioral variables: accuracy and reaction time using Spearman’s rank correlation due to the non-parametric nature of the data. Correlations were assessed separately for each behavioral variable across “Frequency” (theta, alpha, beta), “Time Phases” (approach, early decision, feedback), and “Cluster” (3, 7, 10, 12, 13). *P*-values were adjusted using the Benjamini–Hochberg method to control for multiple comparisons.

## Results

### Behavioral results

**Fig. 3:**
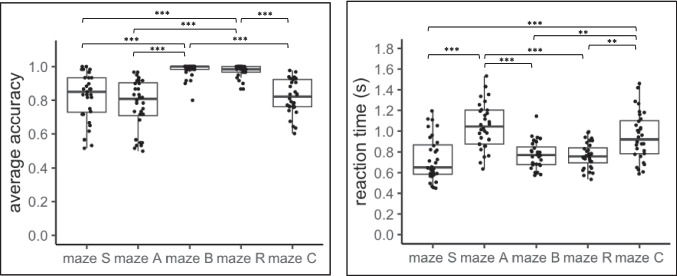
The mean accuracy (**left**) and reaction time (**right**) across trips on mazes. Each dot represents a person. Statistical significance is denoted as *** for *p* <.001, ** for *p* <.01 and * for *p* <.05, after *p*-value correction using the Bonferroni method

The Friedman tests showed significant differences across mazes in both accuracy (χ^2^(4) = 76.39, *p* <.001, W = 0.597) and reaction time (χ^2^(4) = 65.6, *p* <.001, W = 0.513). Figure 3 presents the mean accuracy and reaction time of participants across testing trips for each maze, along with significant pairwise comparisons revealed by Dunn’s tests after *p*-value correction. Detailed statistical outcomes, including *p*-values and effect sizes, are summarized in OSM Table 1. In addition, behavioral results for accuracy and reaction time for each trip from each maze are shown in OSM Fig. 2. Specifically, accuracy was higher in mazes B and R compared to mazes S, A, and C, while reaction time was higher in mazes A and C compared to mazes S, B, and R across trips. No significant differences were observed between mazes B and R, and between mazes S, A, and C regarding accuracy, or between mazes A and C, and between mazes S, B, and R regarding reaction time.

### EEG results

#### Brain clusters

Our cluster identification procedure yielded 13 clusters, each containing ICs from at least 17 of 30 participants (over 50%). These clusters were used to estimate source locations corresponding to distinct brain regions, presented as approximate coordinates that reflect similar activation patterns rather than precise functional localization. A summary of the source localization results for each cluster is provided in Table 3.

**Table 3:** Estimated brain localization of 13 source clusters

Cluster	Region	x, y, z	Brodmann areas	Hemisphere	Near region	Sample size
1	Frontal	−20, 31, 46	BA 8	Left	Superior frontal gyrus	18
2	Frontal	10, 43, 46	BA 8	Right	Superior frontal gyrus	19
3	Frontal	−35, −3, 56	BA 6	left	Middle frontal gyrus	24
4	Frontal	41, 0, 54	BA 6	right	Middle frontal gyrus	23
5	Frontal	−2, −28, 70	BA 6	left	Medial frontal gyrus	27
6	Frontal	5, 8, 61	BA 6	right	Medial frontal gyrus	17
7	Limbic	0, −3, 23	BA 24	left	Cingulate gyrus	19
8	Parietal	39, −27, 45	BA 40	right	Postcentral gyrus	21
9	Parietal	−27, −49, 35	BA 7	left	Precuneus	18
10	Parietal	8, −48, 45	BA 7	right	Precuneus	25
11	Occipital	−38, −69, 7	BA 19	left	Middle occipital gyrus	18
12	Temporal	37, −58, 18	BA 19	right	Middle temporal gyrus	21
13	Occipital	1, −74, 22	BA 18	right	Cuneus	23

*Note.* The clusters were labeled according to their nearest gray-matter regions based on the x, y, z coordinates of each cluster’s centroid, determined using the Talairach Client (Lancaster et al., 2000) and expressed in terms of approximate Brodmann areas (BAs)

#### Time–frequency results

**Fig. 4:**
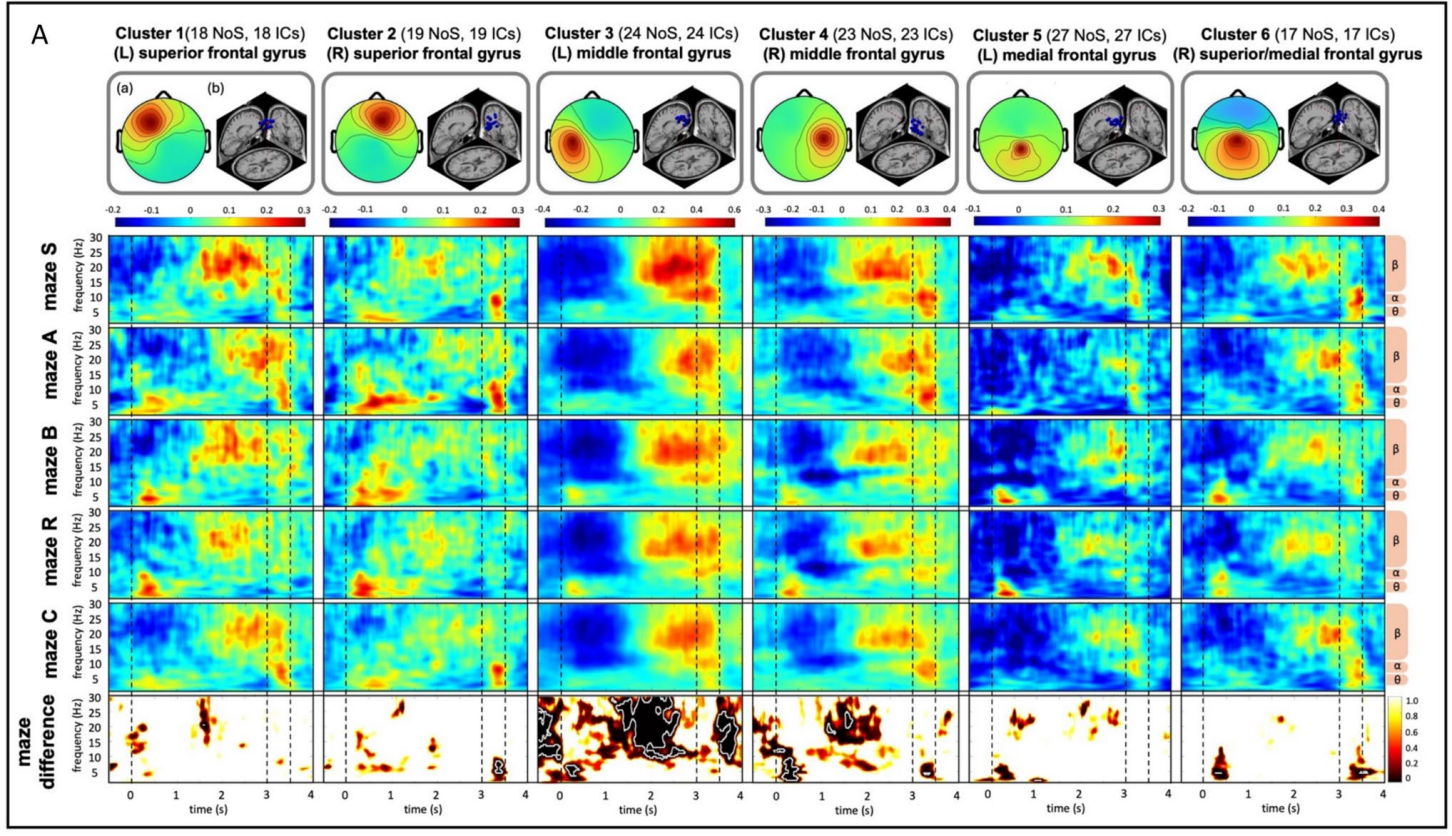

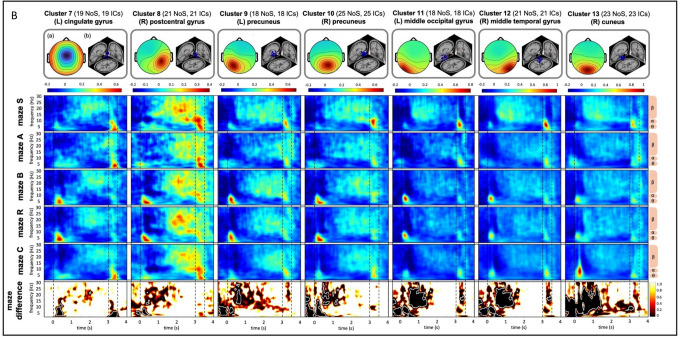
Clustering results of brain-related independent components across participants. (**A**) Clusters 1–6: activity in frontal lobe. (**B**) Clusters 7–13: activity in posterior lobe. “NoS” refers to the number of subjects, and “ICs” denotes the number of independent components included in each cluster. In the top row, (a) presents the EEG scalp map, blue dots in (b) shows individual EEG-dipole source locations, and the red dot in (b) indicates the average dipole source location across all subjects, aligned with a standard MRI template. The next five rows illustrate the z-scores of the averaged time–frequency data, separately for each maze. The bottom row represents differences between mazes (1 = no difference, 0 = strong difference, as per bar on the right side), with regions of significant differences after *p*-value correction outlined in white. Dashed vertical lines delimit the approach (−0.5 s to 0 s), decision (0 s to + 3 s) and feedback (+ 3 s to + 3.5 s) phases

Figure 4 displays the time–frequency activity across the entire epoch from −0.5 s to + 4 s within the 0.5- to 30-Hz frequency range, separated by maze and derived from the defined clusters. Significant differences between mazes were observed across all clusters, varying by time phase and frequency band (see the bottom row: maze difference of Fig. 4, where maze differences that are significant are highlighted by white lines; Fig. 4A shows clusters in frontal regions, and Fig. 4B shows clusters in posterior regions).

All post hoc pairwise comparisons for each cluster are presented in OSM Fig. 3, summarizing significant differences between mazes, separated by those relying on the same reference frame and those that do not. Four frontal clusters showed no significant differences in pairwise comparisons, whereas the remaining two frontal and five posterior clusters exhibited significant differences in some comparisons.

**Fig. 5:**
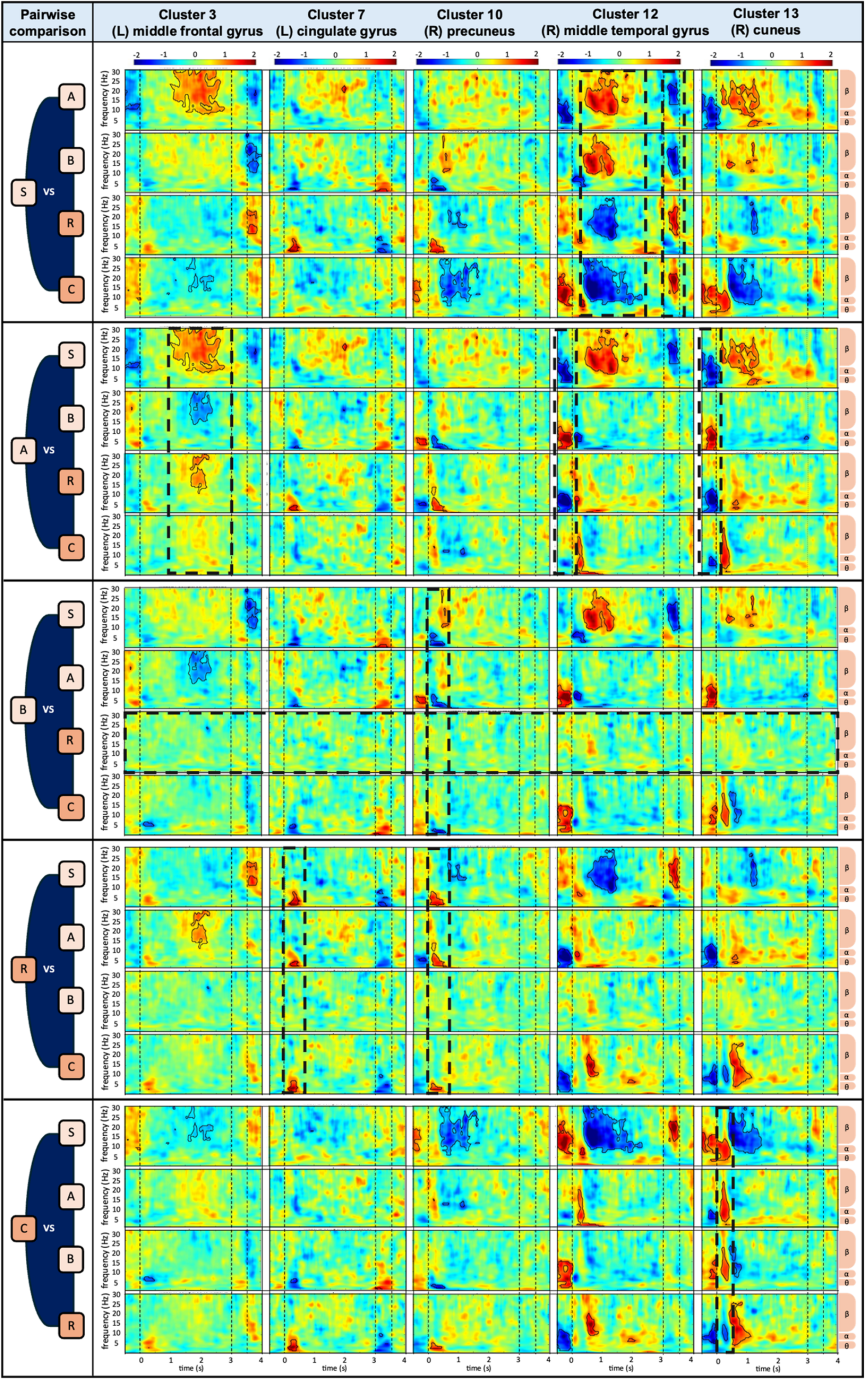
Post hoc pairwise comparisons of each maze to other four for five selected clusters. Each row block represents a maze compared to the other four. The color indicates the magnitude of the effect size using Cohen’s d as shown by the bar at the top. Regions with significant differences after *p*-value correction are highlighted with black outlines. Dashed boxes highlight regions where a maze exhibits a similar significant pattern across maze comparisons. Dashed vertical lines mark the approach (−0.5 s to 0 s), decision (0 s to + 3 s), and feedback (+ 3 s to + 3.5 s) phases. Note that mazes S, A, and B (light orange) rely on the egocentric reference frame, while mazes R and C (dark orange) rely on both egocentric and allocentric reference frame for decision making

To provide a focused overview of maze-specific activation patterns, we presented five representative clusters that correspond to major brain regions: frontal (cluster 3, left middle frontal gyrus), limbic (cluster 7, left cingulate gyrus), parietal (cluster 10, right precuneus), temporal (cluster 12, right middle temporal gyrus), and occipital (cluster 13, right cuneus). The clusters were selected based on the presence of larger significant regions involved in the decision-making process in Fig. 4. In cases where bilateral activity was observed, the hemisphere with a higher number of contributing ICs was chosen. Figure 5 illustrates pairwise maze comparisons between each maze and the other four mazes based on the five selected clusters. Significant differences were observed in specific clusters and time phases across the comparisons, except between mazes B and R (see Fig. 5c, horizontal dashed box indicating no significant difference between these two mazes). The consistently observed significant differences, reflecting distinct activation patterns for each maze, are summarized in Table 4.

**Table 4:** Summary of differences in comparisons between a maze and the other mazes

Maze	Cluster	Region	Frequency	Time phase	Differences	Activity
S (Fig. 5a)	12	Temporal	Beta	Later decision	All mazes	Higher
	12	Temporal	Beta	Feedback	All mazes	Lower
A (Fig. 5b)	3	Frontal	Beta	Later decision	Mazes S, B, R	Lower
	12	Temporal	Theta-alpha	Approach	Mazes S, B, R	Higher
	13	Occipital	Theta-alpha	Approach	All mazes	Higher
B (Fig. 5c)	10	Parietal	Theta	Early decision	Mazes S, A	Higher
R (Fig. 5d)	7	Limbic	Theta	Early decision	Mazes S, A, C	Higher
	10	Parietal	Theta	Early decision	Mazes S, A, C	Higher
C (Fig. 5e)	13	Occipital	Theta-alpha	Early decision	All mazes	Higher

*Note.* The first column indicates the primary maze being compared against the other mazes. Significant differences between maze pairs are listed in the “differences” column

#### Mean power activity results

**Fig. 6:**
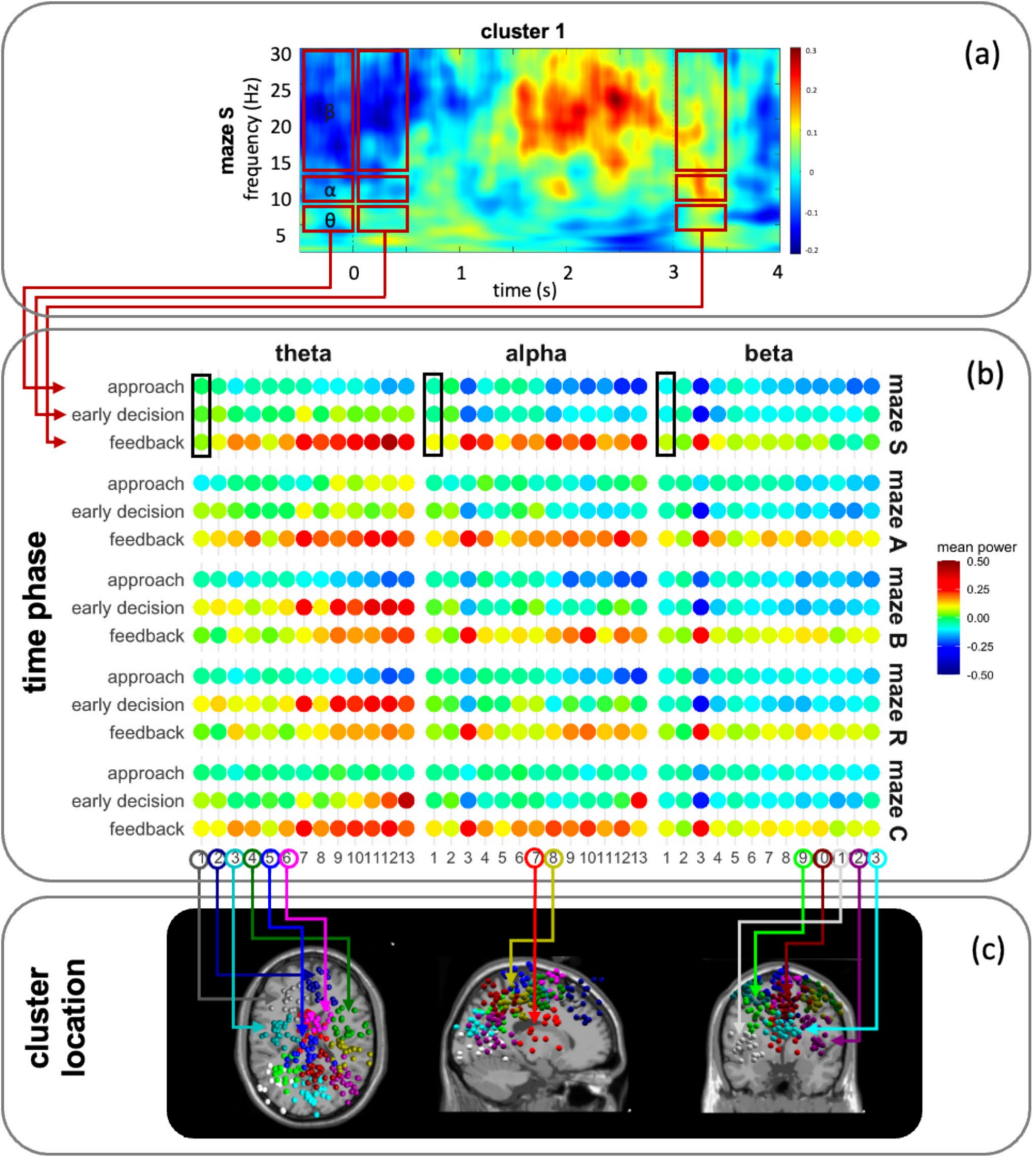
Illustration of mean power activity extraction from time–frequency data and their results with corresponding ICs location of each cluster. (**a**) Example of the time–frequency data from Fig. 4 (maze S, cluster 1), with the color bar representing z-scores. Red boxes indicate the segments selected for calculating mean EEG power. (**b**) Mean EEG power (z-scores) shown as dots for each cluster, separated by time phase, maze, and frequency band; black boxes highlight values from the exemplary cluster 1. (**c**) Illustration of the IC locations, color-coded by cluster number as indicated

Figure 6 illustrates the average power activity from Fig. 4, segmented by specific time phases (approach: −0.5 s to 0 s; early decision: 0 s to + 0.5 s; feedback: + 3 s to + 3.5 s), separately for each cluster, maze and frequency band. In particular, Fig. 6b highlights a noticeable increase in theta activity during the early decision and feedback phases, especially within posterior regions (clusters 7–13), compared to the approach phase. Similarly, alpha and beta power were generally higher during the feedback phase than during the approach and early decision phases. However, increased alpha activity was observed in both anterior and posterior regions (i.e., clusters 3–13), depending on the specific region. These results indicate that neural activity within the same regions varies substantially across different phases of the decision-making process.

The Type III ANOVA outcomes across mazes, derived from the linear mixed-effects models, are summarized in Table 5. Significant main effects were observed for maze, time phase, and cluster, as well as for their two-way interactions, with the exception of the Maze × Cluster interaction in the theta and beta bands. Moreover, a significant three-way interaction (Maze × Time Phase × Cluster) was indeed observed across all three frequency bands. These findings indicate that brain activation patterns differ between mazes, and these differences are modulated by both the temporal phase and the spatial location of neural activity.

**Table 5:** Type III ANOVA outcome across mazes for each frequency band

Effect	Sum Sq	numDF	denDF	F	*p*-value	*p*-adj	η^2^_p_
**Theta**							
Maze	0.41	4	3871.3	5.64	<.001	** <.001**	0.01
Time phase	36.03	2	3871.3	1000.24	<.001	** <.001**	0.34
Cluster	5.56	12	3893.7	25.71	<.001	** <.001**	0.07
Maze x Time phase	10.43	8	3871.3	72.37	<.001	** <.001**	0.13
Maze x Cluster	1.04	48	3871.3	1.20	.159	.159	0.01
Time phase x Cluster	7.49	24	3871.3	17.33	<.001	** <.001**	0.10
Maze x Time phase x Cluster	6.43	96	3871.3	3.72	<.001	** <.001**	0.08
**Alpha**							
Maze	1.00	4	3871.0	15.13	<.001	** <.001**	0.02
Time phase	45.99	2	3871.0	1399.45	<.001	** <.001**	0.42
Cluster	0.83	12	3888.6	4.21	<.001	** <.001**	0.01
Maze x Time phase	2.81	8	3871.0	21.35	<.001	** <.001**	0.04
Maze x Cluster	1.61	48	3871.0	2.04	<.001	** <.001**	0.02
Time phase x Cluster	10.62	24	3871.0	26.93	<.001	** <.001**	0.14
Maze x Time phase x Cluster	4.46	96	3871.0	2.83	<.001	** <.001**	0.07
**Beta**							
Maze	0.67	4	3870.9	24.59	<.001	** <.001**	0.02
Time phase	35.76	2	3870.9	2630.23	<.001	** <.001**	0.58
Cluster	1.31	12	3886.6	16.01	<.001	** <.001**	0.05
Maze x Time phase	0.62	8	3870.9	11.42	<.001	** <.001**	0.02
Maze x Cluster	0.42	48	3870.9	1.28	.092	.097	0.02
Time phase x Cluster	9.77	24	3870.9	59.86	<.001	** <.001**	0.27
Maze x Time phase x Cluster	0.94	96	3870.9	1.45	.003	.**004**	0.03

*Note. p.*adj denotes *p*-values adjusted for multiple comparisons using the Benjamini–Hochberg method

Detailed Type III ANOVA outcomes from the expanded subset analysis of pairwise maze comparisons, including adjusted *p*-values and effect sizes, are presented in OSM Table 2. Table 6 summarizes the three-way interaction effects, highlighting spatiotemporal pattern differences between maze pairs. In the theta band, all comparisons showed significant differences except between mazes B and R. In the alpha band, significant effects were observed in comparisons involving maze A or maze C against the others (mazes S, B, and R). In the beta band, only comparisons between maze S and all other mazes (mazes A, B, R, and C) reached significance. These findings are consistent with the visualized results from Fig. 5, which revealed similar activation patterns between mazes B and R, while the remaining mazes displayed distinct activation characteristics.

**Table 6:** Summary of Type III ANOVA outcomes for three-way interaction effects in pairwise maze comparisons

Pairwise comparison	Maze x Time phase x Cluster		
	Theta	Alpha	Beta
**Pairs relying on the same reference frame**			
Maze S vs. A	***	***	***
Maze S vs. B	***	n.s.	**
Maze A vs. B	***	***	n.s.
Maze R vs. C	***	***	n.s.
**Pairs relying on not the same reference frame**			
Maze R vs. S	***	n.s.	***
Maze R vs. A	***	***	n.s.
Maze R vs. B	n.s.	n.s.	n.s.
Maze C vs. S	***	***	***
Maze C vs. A	***	***	n.s.
Maze C vs. B	***	***	n.s.

*Note.* Asterisks indicate significance levels after *p*-value correction using the Benjamini–Hochberg method: ***p <.001, **p <.01, *p <.05 and “n.s.” indicates non-significant

#### Neural-behavioral correlation results

Table 7 summarizes the Spearman correlation results between brain power activity and behavioral outcome (accuracy and reaction time), stratified by time phases and frequency bands across five clusters. Detailed statistical outcomes are provided in OSM Tables 3 and 4, with corresponding correlation plots shown in OSM Figs. 4 and 5.

**Table 7:** Summary of Spearman correlation between EEG power and behavior performance

Time point	Cluster	Region	Theta		Alpha		Beta	
			Acc	RT	Acc	RT	Acc	RT
Approach	3	Frontal	n.s.	+ *	-**	+ ***	n.s.	+ ***
	7	Limbic	n.s.	n.s.	n.s.	n.s.	n.s.	+ **
	10	Parietal	-***	+ ***	n.s.	+ **	n.s.	n.s.
	12	Temporal	-***	+ ***	-***	+ ***	n.s.	+ **
	13	Occipital	-***	+ ***	-***	+ ***	n.s.	n.s.
Early decision	3	Frontal	+ **	n.s.	n.s.	n.s.	n.s.	n.s.
	7	Limbic	+ **	-*	n.s.	n.s.	n.s.	+ *
	10	Parietal	+ **	n.s.	n.s.	n.s.	n.s.	n.s.
	12	Temporal	+ **	n.s.	n.s.	n.s.	n.s.	n.s.
	13	Occipital	n.s.	+ **	n.s.	+ **	n.s.	n.s.
Feedback	3	Frontal	-*	n.s.	n.s.	n.s.	n.s.	+ **
	7	Limbic	-***	n.s.	-*	n.s.	n.s.	+ ***
	10	Parietal	-*	+ **	n.s.	n.s.	n.s.	+ ***
	12	Temporal	-**	n.s.	n.s.	+ *	n.s.	+ **
	13	Occipital	n.s.	n.s.	n.s.	n.s.	n.s.	+ *

*Note.* Acc represents accuracy, and RT represents reaction time. A “+” indicates a significant positive correlation, a “-” indicates a significant negative correlation, and “n.s.” denotes a non-significant result. Asterisks indicate significance levels after *p*-value correction using the Benjamini–Hochberg method: ***p <.001, **p <.01, and *p <.05

Regarding correlations with accuracy, theta and alpha power showed partially significant negative associations during the approach and feedback phases across clusters, indicating that higher accuracy was associated with lower power activity. In contrast, a significant positive correlation was observed for theta power during the early decision phase, where higher accuracy corresponded with increased theta activity, while no such effect was found for alpha power. Furthermore, beta power showed no significant correlation with accuracy across any time phase or cluster, suggesting that beta activity was not related to task accuracy.

Regarding correlations with reaction time, theta, alpha, and beta power showed partially significant positive associations across all three phases, varying by cluster. This suggests that higher power in these frequency bands was generally related to longer reaction times. An exception was found for theta power during the early decision phase in cluster 7, where a significant negative correlation indicated that lower theta activity was associated with longer reaction times.

## Discussion

The present study investigated the spatiotemporal dynamics of brain activity during wayfinding, with a focus on decision making at intersections. Using a within-subject design, participants had to find their way through five virtual mazes, each designed to be associated with one particular decision-making strategy. EEG was used to capture cortical activity with high temporal resolution. We hypothesized that each strategy would be associated with a somewhat different spatiotemporal pattern of cortical activation, due to its unique cognitive processing demands, regardless of whether a purely egocentric or a combined egocentric-allocentric frame of reference was involved.

Behavioral results showed higher accuracy for the beacon and relative location strategy (mazes B and R), and lower accuracy for the serial order, associative cue, and cognitive map strategy (mazes S, A, and C). Reaction times were shorter for the serial order, beacon, and relative location strategy compared to the associative cue and cognitive map strategy. These findings replicate previous studies (Bock & Huang, 2024; Bock et al., 2023, 2024). Overall, strategies with low memory demand, which rely primarily on egocentric judgments of visible objects, yield higher accuracy and faster decisions than those requiring greater memory load, such as sequential memory, associative memory, or the formation of a cognitive map. Additionally, strategies that allow decisions to be made in advance result in shorter response times than those requiring real-time and memory-demanding decisions at intersections.

Regarding our EEG data results, we identified clusters of dipoles distributed across several brain regions. Frontal clusters included areas near the superior frontal gyrus (clusters 1–2, BA 8) and the middle and medial frontal gyri (clusters 3–6, BA 6). Despite EEG source localization offering only approximate spatial precision, the retrieved regions are consistent with previous findings on the neural processes underlying wayfinding decisions. The prefrontal cortex has been implicated in decision making, goal tracking, and route planning (He et al., 2024; Patai & Spiers, 2021). The middle frontal gyrus is linked to spatial attention, working memory, and cognitive control, while the supplementary motor area supports motor planning and sensorimotor integration during navigation (Cona & Scarpazza, 2019; Cona & Semenza, 2017).

Posterior clusters were located near the cingulate gyrus of the limbic lobe (cluster 7, BA 24); the postcentral gyrus (cluster 8, BA 40) and the precuneus (cluster 9–10, BA 7) in the parietal lobe; the middle temporal gyrus (cluster 11, BA 19) of the temporal lobe; and the middle occipital gyrus (cluster 12, BA 19) and cuneus (cluster 13, BA 18) of the occipital lobe. These retrieved clusters are consistent with prior EEG studies that have identified similar regions during active wayfinding tasks (e.g., Delaux et al., 2021; Plank et al., 2010). These activation patterns are consistent with the established roles of these regions in spatial processing and integration. Specifically, the anterior cingulate cortex is associated with route re-evaluation, error monitoring in spatial orientation, and regulation of cognitive effort (Patai & Spiers, 2021). The postcentral gyrus supports spatial awareness through sensory processing of environmental stimuli (Sack, 2009) and, alongside the supramarginal gyrus, facilitates spatial attention and memory (Cona & Scarpazza, 2019; Sneider et al., 2018). The precuneus plays a key role in integrating visuospatial information, self-referential spatial updating, and memory retrieval (Cona & Scarpazza, 2019; Dordevic et al., 2022; Ghaem et al., 1997). Additionally, the middle temporal gyrus, middle occipital gyrus, and cuneus are involved in visual-spatial information processing critical to active wayfinding (Ohnishi et al., 2006; Palejwala et al., 2021; Spiers & Maguire, 2006).

Our time–frequency decomposition findings align with previous evidence showing consistent activation of the superior frontal gyrus across all five strategies, indicating a general role of the prefrontal cortex in wayfinding decisions. In contrast, strategy-specific differences were observed in other frontal regions (e.g., middle and medial frontal gyri) and posterior areas, reflecting the particular spatial strategies or types of information utilized (Hartley et al., 2003; Spiers & Maguire, 2008).

In detail, we found that the serial order strategy (maze S) was associated with increased beta activity during the later decision phase and decreased activity during the feedback phase in temporal regions. This pattern likely reflects reliance on pre-learned directional sequences, requiring proactive maintenance and tracking of turn order (e.g., left–right-left). These findings are consistent with prior research linking beta oscillations to sequence processing in working memory and top-down control, facilitating prediction generation, updating of internal representations, and preparation for upcoming actions (Spitzer & Haegens, 2017).

We further found that the associative cue strategy (maze A) was characterized by a prolonged decrease in frontal beta activity during the later decision phase, and increased theta-alpha activity in the temporal-occipital regions during the approach phase. This pattern likely reflects reliance on externally guided cues and the high memory demands of associative retrieval, requiring the matching of current visual input with stored cue-direction associations. These results align with previous findings linking decreased frontal beta activity to working memory updating or ongoing cognitive effort (Spitzer & Haegens, 2017), and increased occipital theta-alpha activity to visual processing and scene recognition (Cheng et al., 2022; Delaux et al., 2021; Du et al., 2023; Naveilhan et al., 2023).

Notably, we observed a similar activation pattern for the beacon and relative location strategy (mazes B and R), characterized by increased theta activity in the limbic-parietal regions during the early decision phase. This likely reflects a shared reliance on an egocentric, visual cue-piloting wayfinding process, involving direction judgments based on widely visible cues and the selection of an unblocked route. While the relative location strategy (maze R) involves additional distance estimation, the task demands may have been too subtle to notice distinct neural differences. This pattern aligns with prior findings linking the parietal theta activity (i.e., posterior cingulate, precuneus) to landmark-based wayfinding and goal-direction decision making (Delaux et al., 2021; Lin et al., 2022; Taherigorji, 2021).

Additionally, we found that the cognitive map strategy (maze C) uniquely elicited elevated theta-alpha activity in the parieto-occipital region during early decision making, possibly reflecting dynamic spatial updating and alignment within an internal spatial map of the environment. This finding aligns with previous studies reporting that theta activity in these regions (i.e., retrosplenial cortex) supports spatial updating, including head-direction changes and real-time integration of egocentric and allocentric spatial representations (Delaux et al., 2021; Do et al., 2021; Gramann et al., 2021; Lin et al., 2015; Nguyen et al., 2014).

Our spatiotemporal synthesis of mean EEG power across time × frequency × cluster dimensions partially confirms our hypothesis that each strategy is associated with distinct neural activation patterns, as revealed in the above visualization results. Particularly, we found that in the theta band, beacon and relative location strategy (mazes B and R) exhibited similar spatiotemporal patterns, distinct from those of the serial order, associative cue, and cognitive map strategy (mazes S, A, and C). This is in line with evidence that theta activity is central to spatial processing (McLaren-Gradinaru et al., 2025) and is sensitive to different cognitive control and process during navigation (Gramann et al., 2010; Klimesch, 1999). In the alpha band, the associative cue and cognitive map strategy showed distinct patterns compared to the other four. This is again consistent with the role of alpha oscillations in visual processing and visual-spatial integration, particularly under contexts requiring complex cue interpretation, rather than non-cue or cue-piloting navigation (Delaux et al., 2021; Lin et al., 2022; Taherigorji, 2021). In the beta band, only the serial order strategy showed a distinct pattern compared to the other four strategies, suggesting that beta activity may reflect not only motor processing (Mikropoulos, 2001) but also internal reference and prediction-based navigation (Meijer et al., 2016; Spitzer & Haegens, 2017; Tzagarakis et al., 2015).

Finally, our neural-behavioral correlation analysis showed that lower accuracy and longer reaction time were partly linked to increased theta, alpha, and beta activity during the approach and feedback phases, across different brain regions. This indicates greater neural effort during higher cognitively demanding conditions (e.g., mazes A and C), both in processing the environment prior to decision making and in evaluating feedback and reinforcement (i.e., correct responses). This finding aligns with previous studies that increased theta activity is linked to higher cognitive effort, feedback monitoring, and reward processing (Chrastil et al., 2022; Klimesch, 1999; Lin et al., 2022). Increased alpha power reflects heightened visual-spatial attention and inhibitory control necessary for complex visual processing (Klimesch, 2012). Furthermore, increased beta activity is related to prolonged motor preparation (Schapkin et al., 2020) and the processing of positive reinforcement in tasks with higher cognitive demands (HajiHosseini & Holroyd, 2015; Marco-Pallarés et al., 2015).

In addition, we observed that higher accuracy and shorter reaction times (i.e., lower cognitive demand) were associated with increased theta activity in frontal, limbic, parietal, and temporal regions during the early decision-making phase. In contrast, longer reaction times (i.e., higher cognitive demand) were correlated with increased theta and alpha activity in the occipital region. These opposing trends suggest that elevated theta activity reflects different cognitive demands depending on the brain region, highlighting distinct spatial processing mechanisms, as we revealed. For example, increased theta in frontal-limbic-parietal regions, which have been implicated in successful cue-piloting and goal-directed wayfinding, may indicate more efficient, lower-demand cognitive control (Chrastil et al., 2022). Conversely, heightened theta and alpha activity in parietal-occipital areas, which we found to be engaged in spatial updating, likely reflects greater neural effort required for successful wayfinding under higher cognitive load (Delaux et al., 2021; Du et al., 2023; White et al., 2012).

Taken together, our findings demonstrate that spatiotemporal brain activation patterns during wayfinding differ according to specific cognitive strategies, rather than fitting seamlessly into a simple egocentric versus allocentric dichotomy. Theta activity in limbic and parietal regions was strongly associated with cue-based piloting and goal-directed wayfinding, which involve the use of widely visible visual cues. Additionally, theta and alpha activity in parietal-occipital regions reflected processes of spatial updating and the integration of egocentric and allocentric information. Alpha activity in temporal-occipital areas was particularly linked to association-based wayfinding guided by nearby visual cues, whereas beta activity was associated with strategies that rely on internal serial order memorization in the absence of external cues. Our findings suggest that the neural correlates of wayfinding strategies are closely tied to the types of spatial information available in the environment and the specific cognitive processes they engage. Thus, the classification of cognitive strategies may be more appropriately based on the presence and type of visual cues, and whether spatial relationship judgments are used in the wayfinding task (see Table 8 for an exemplary categorization).

**Table 8:** Classification of wayfinding strategies based on visual cue type and spatial judgment

		Type of visual cue		
		Absent	Associative cue	Widely visible cue
Spatial Relationship Judgement	No	Serial order memorization	Items memorization/route following	Piloting
	Yes	Self-motion/ path integration	Cognitive map	Relative location

A potential limitation of this study is that participants did not physically navigate through the mazes, resulting in the absence of a natural interplay between visual input, vestibular-proprioceptive feedback, and locomotor control. Previous studies have shown that active walking engages a specific neural activation pattern, reflected particularly in theta oscillations (Delaux et al., 2021; Do et al., 2021), and this activity is likely missing in our data.

Another limitation is the reliance on scalp EEG, which primarily captures cortical surface activity and depends on standardized electrode placements and standard averaged MRI templates. Although EEG source reconstruction methods have been validated (Klug et al., 2022, 2024), this approach may reduce spatial accuracy and warrants cautious interpretation due to the lack of participant-specific imaging data.

In conclusion, this study provides new insights into the cortical spatiotemporal neural dynamics underlying specific cognitive strategies in human wayfinding. Our findings show that cognitive strategies cannot be fully explained by an egocentric-allocentric dichotomy. Instead, cognitive strategies are partially characterized by the types of spatial information available (e.g., internal cues, nearby visual cues, distal visual cues) and the cognitive processes they engage (e.g., goal-directed, item/associative memory, spatial information updating and integration). Distinct patterns of theta, alpha, and beta activity across brain regions correspond to these varied strategies. These results highlight the need for a refined way of classifying wayfinding cognitive strategies that considers both environmental cue availability and mental processes involved. This approach can support future research in the design of spaces by aligning them with how people use available information to navigate.

## Supplementary Information

Below is the link to the electronic supplementary material.

Supplementary file1 (DOCX 3339 kb)

## Data Availability

The datasets generated and/or analyzed during the current study are available from the corresponding author upon reasonable request.

## Code availability

The code for running the experiments and datasets generated and analyzed during the current study are available from the corresponding author on reasonable request.
