# Supplementary material for: Cortical dynamics are differentially associated with decision-making strategies in human wayfinding: An EEG study
Source: Cogn Affect Behav Neurosci. 2025 Nov 6;26(1):120–38. doi: 10.3758/s13415-025-01357-2 (PMC12847099; doi:10.3758/s13415-025-01357-2)
Supplement: Supplementary file 1 — Supplementary file1 (DOCX 3339 kb) [file 13415_2025_1357_MOESM1_ESM.docx]

**Supplementary Materials**

**Supplementary Figure 1**

*Electrode impedance distribution for each participant*

**
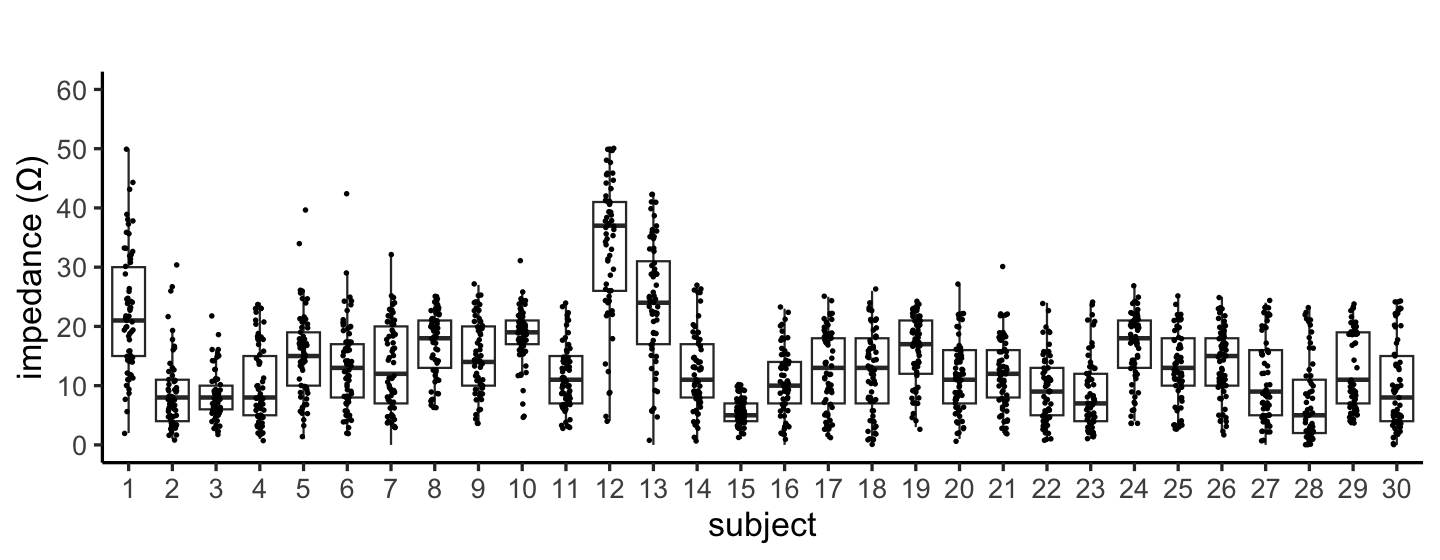
**

*Note.* Each dot represents a channel from a subject.

**EEG data analysis and pre-processing details**

**(1) Filter specifications:**

To minimize slow signal drifts, a temporary high-pass filter with a cutoff frequency of 1.5 Hz was applied. This was implemented using a zero-phase finite impulse response (FIR) filter with a Hamming window, filter order of 1650, and a transition bandwidth of 0.5 Hz, yielding an effective cutoff frequency of approximately 1.25 Hz. This filtering strategy aligns with the recommendations of Klug and Gramann (2021) to enhance data quality for subsequent independent component analysis (ICA).

**Supplementary Figure 2**

*Accuracy (top) and reaction time (bottom) of each trip for each maze*

**
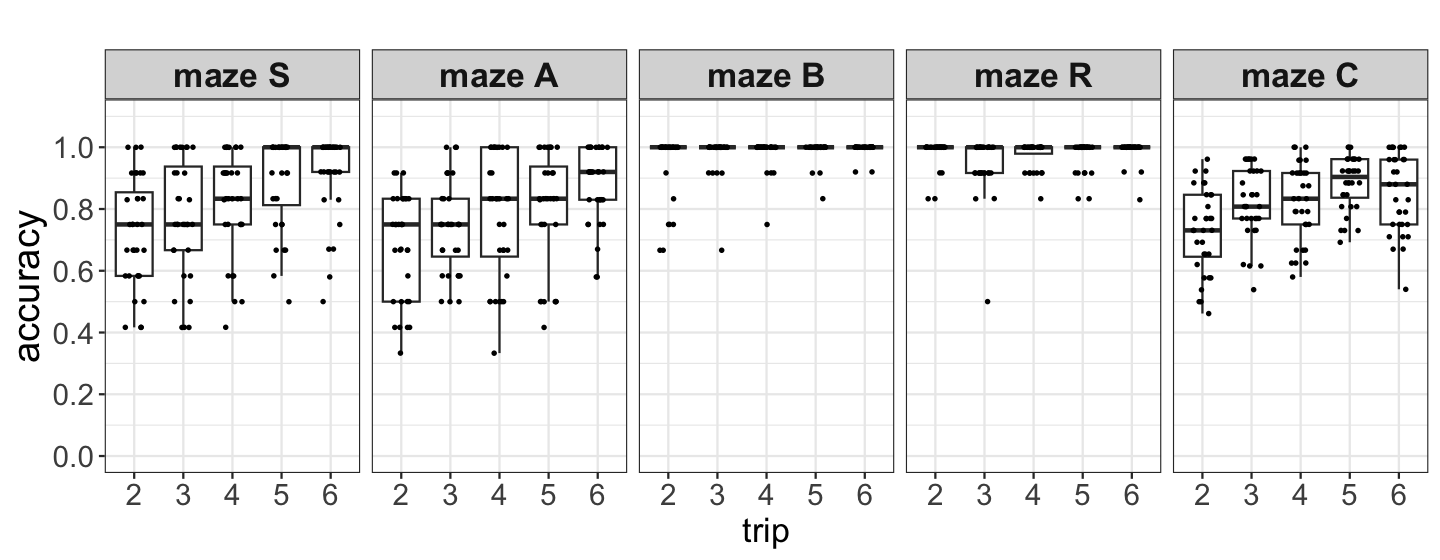
**

**
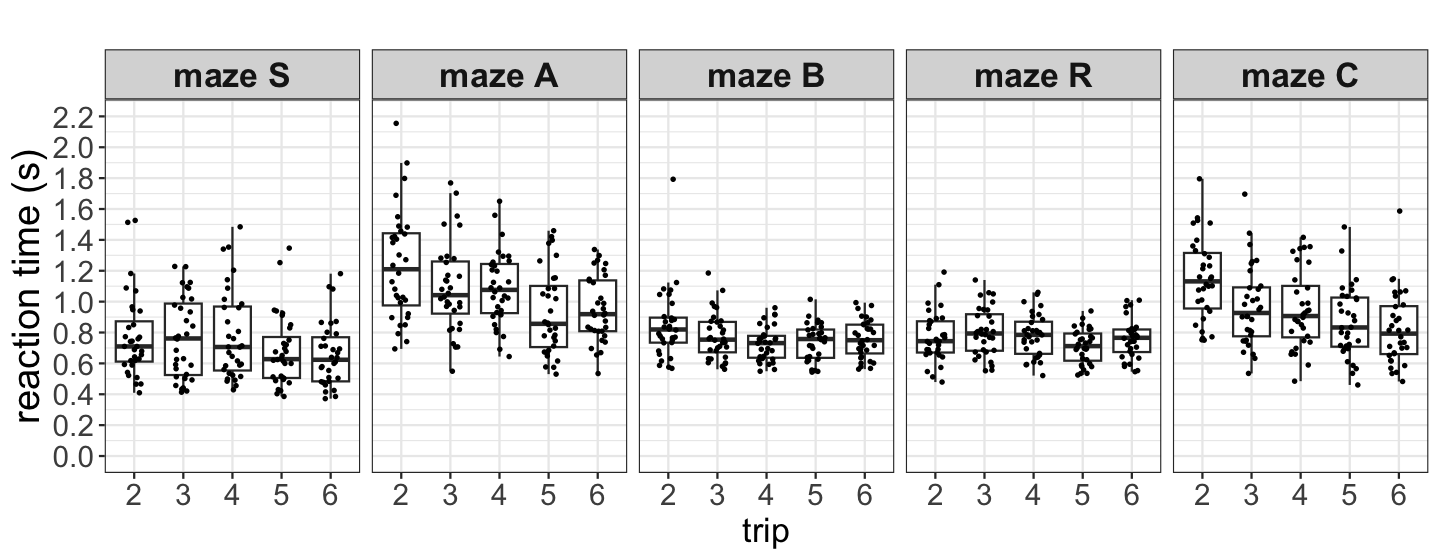
**

*Note.* Each dot represents a person. Trip 1 was externally guided and is therefore not included.

**Supplementary Table 1**

*Dunn’s post-hoc test outcomes for pairwise maze comparisons on accuracy and reaction time*

| **Pairwise**  **Comparison** | **Accuracy** | | | | **Reaction Time** | | | |
| --- | --- | --- | --- | --- | --- | --- | --- | --- |
|  | **Z** | ***p*** | ***p.adj*** | ***r*** | **Z** | ***p*** | ***p.adj*** | ***r*** |
| maze S vs A | -1.27 | .102 | 1.000 | -0.10 | 5.61 | < .001 | **< .001** | 0.44 |
| maze S vs B | 5.61 | < .001 | **< .001** | 0.44 | 0.86 | 0.196 | 1.000 | 0.07 |
| maze S vs R | 5.05 | < .001 | **< .001** | 0.40 | 0.81 | 0.208 | 1.000 | 0.06 |
| maze S vs C | -0.62 | 0.269 | 1.000 | -0.05 | 3.99 | < .001 | **< .001** | 0.32 |
| maze A vs B | -6.88 | < .001 | **< .001** | -0.54 | 4.76 | < .001 | **< .001** | 0.38 |
| maze A vs R | -6.32 | < .001 | **< .001** | -0.50 | 4.80 | < .001 | **< .001** | 0.38 |
| maze A vs C | -0.65 | 0.257 | 1.000 | -0.05 | 1.62 | 0.052 | 0.525 | 0.13 |
| maze B vs R | 0.56 | 0.288 | 1.000 | 0.04 | 0.04 | 0.484 | 1.000 | 0.00 |
| maze B vs C | 6.22 | < .001 | **< .001** | 0.49 | -3.14 | < .001 | **0.009** | -0.25 |
| maze R vs C | -5.66 | < .001 | **< .001** | -0.45 | 3.18 | < .001 | **0.007** | 0.25 |

*Note. p.adj* denotes *p*-values adjusted for multiple comparisons using the Bonferroni method and *r* represents the effect size calculated using the rank-biserial correlation coefficient.

**Supplementary Figure 3**

*Post-hoc results of pairwise comparisons with effect size (A) Cluster 1-6: frontal lobe (B) Cluster 7-13: limbic, temporal, parietal, occipital lobes*

A


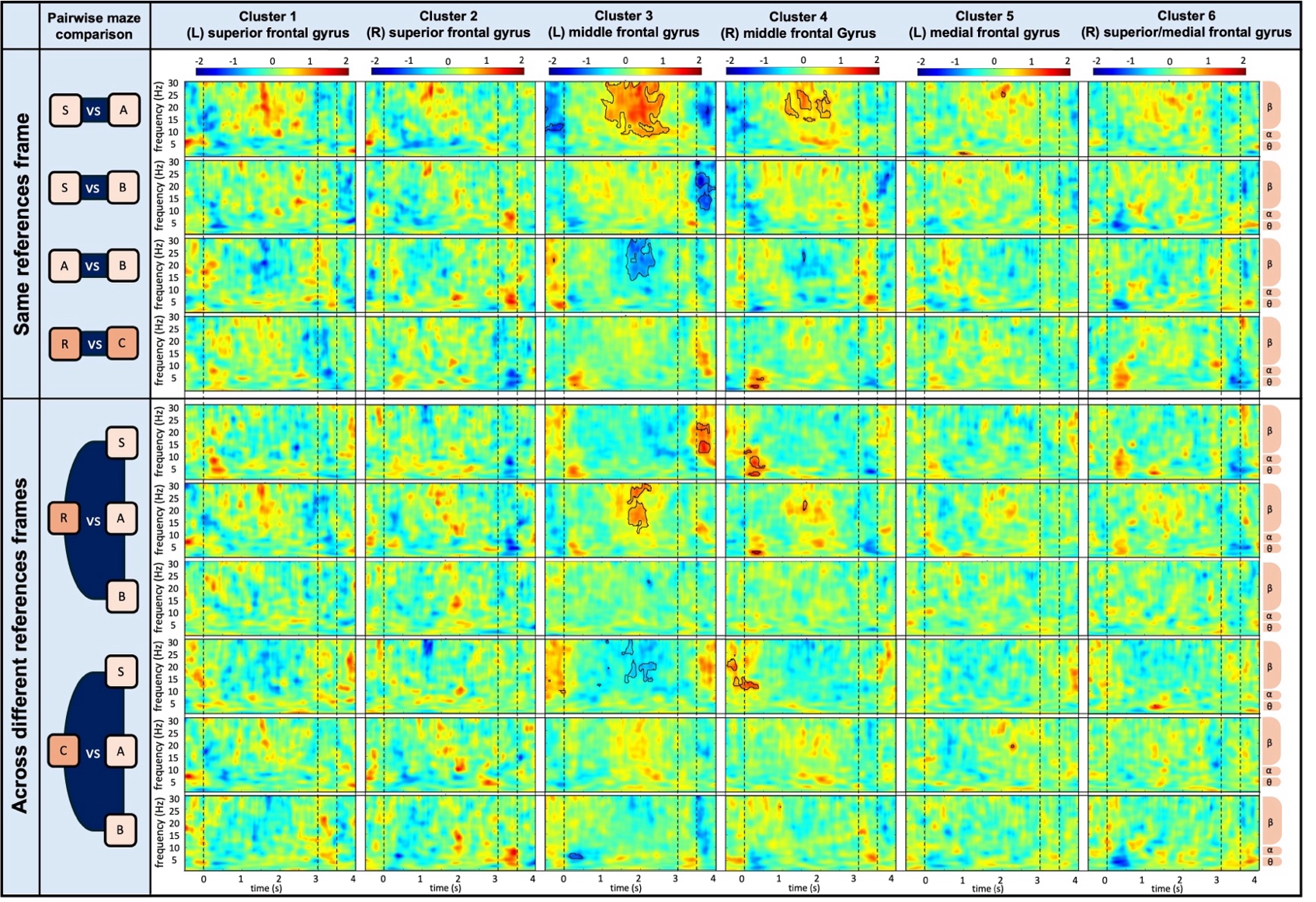


*
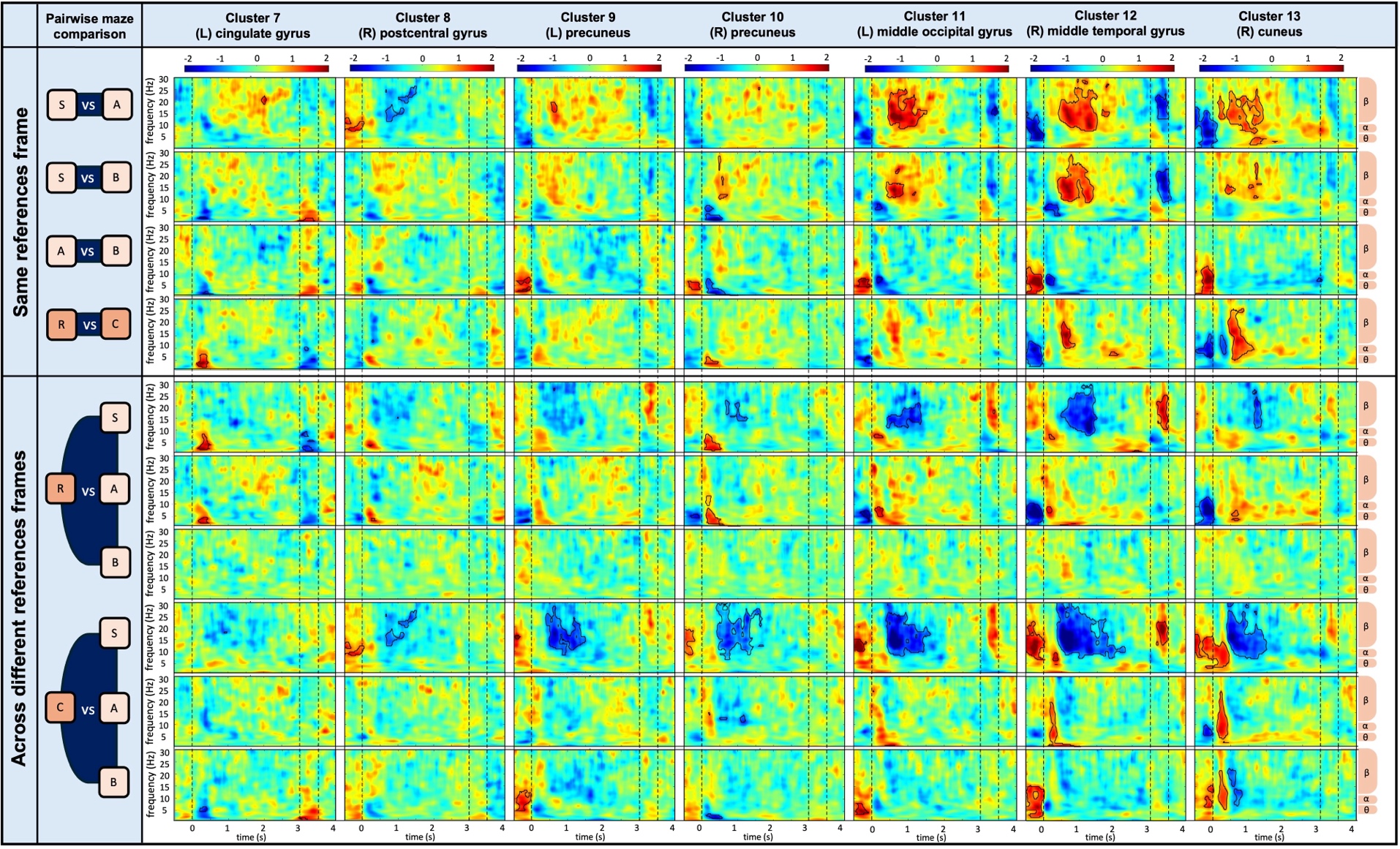
*

B

*Note*. The color represents the magnitude of the effect size using Cohen’s d, with the first maze compared to the second, as indicated by the bar at the top. Regions with significant differences after *p*-value correction are outlined in black. Dashed vertical lines mark the approach (-0.5 s to 0 s), decision (0 s to +3 s), and feedback (+3 s to +3.5 s) phases. Note that maze S, A, and B (light orange) rely on the egocentric reference frame, while maze R and C (dark orange) rely on both egocentric and allocentric reference frames for decision-making.

**Supplementary Table 2**

*Type III ANOVA outcome from linear mixed-effects model of pairwise maze comparisons*

| **Effect** | **Theta** | | | | | | | **Alpha** | | | | | | | **Beta** | | | | | | |
| --- | --- | --- | --- | --- | --- | --- | --- | --- | --- | --- | --- | --- | --- | --- | --- | --- | --- | --- | --- | --- | --- |
|  | **Sum**  **Sq** | **Num**  **DF** | **Den**  **DF** | **F** | ***p*-value** | ***p*-adj.** | **η²_p_** | **Sum**  **Sq** | **Num**  **DF** | **Den**  **DF** | **F** | ***p*-value** | ***p*-adj.** | **η²_p_** | **Sum**  **Sq** | **Num**  **DF** | **Den**  **DF** | **F** | ***p*-value** | ***p*-adj.** | **η²_p_** |
| **Pairs relying on the same reference frame** | | | | | | | | | | | | | | | | | | | | | |
| **maze S vs A** |  |  |  |  |  |  |  |  |  |  |  |  |  |  |  |  |  |  |  |  |  |
| Maze | 0.23 | 1 | 1531.4 | 12.30 | < .001 | **.001** | 0.01 | 0.43 | 1 | 1530.8 | 23.42 | < .001 | **< .001** | 0.02 | 0.50 | 1 | 1530.5 | 62.36 | < .001 | **< .001** | 0.04 |
| Time phase | 16.18 | 2 | 1531.4 | 427.47 | < .001 | **< .001** | 0.36 | 25.12 | 2 | 1530.8 | 678.45 | < .001 | **< .001** | 0.47 | 14.12 | 2 | 1530.5 | 885.27 | < .001 | **< .001** | 0.54 |
| Cluster | 2.28 | 12 | 1551.9 | 10.04 | < .001 | **< .001** | 0.07 | 0.62 | 12 | 1547.1 | 2.77 | < .001 | **.001** | 0.02 | 0.92 | 12 | 1547.1 | 9.60 | < .001 | **< .001** | 0.07 |
| Maze x Time phase | 0.87 | 2 | 1531.4 | 23.04 | < .001 | **< .001** | 0.03 | 0.79 | 2 | 1530.8 | 21.28 | < .001 | **< .001** | 0.03 | 0.48 | 2 | 1530.5 | 30.01 | < .001 | **< .001** | 0.04 |
| Maze x Cluster | 0.24 | 12 | 1531.4 | 1.07 | .382 | .435 | 0.01 | 0.51 | 12 | 1530.8 | 2.29 | 0.007 | **.010** | 0.02 | 0.13 | 12 | 1530.5 | 1.34 | .191 | .228 | 0.01 |
| Time phase x Cluster | 2.67 | 24 | 1531.4 | 5.88 | < .001 | **< .001** | 0.08 | 4.09 | 24 | 1530.8 | 9.21 | < .001 | **< .001** | 0.13 | 4.16 | 24 | 1530.5 | 21.72 | < .001 | **< .001** | 0.25 |
| Maze x Time phase x Cluster | 1.94 | 24 | 1531.4 | 4.27 | < .001 | **< .001** | 0.06 | 1.42 | 24 | 1530.8 | 3.20 | < .001 | **< .001** | 0.05 | 0.62 | 24 | 1530.5 | 3.23 | < .001 | **< .001** | 0.05 |
| **maze S vs B** |  |  |  |  |  |  |  |  |  |  |  |  |  |  |  |  |  |  |  |  |  |
| Maze | 0.03 | 1 | 1531.0 | 1.76 | .184 | .221 | < 0.01 | 0.02 | 1 | 1531.4 | 1.45 | .229 | .269 | < 0.01 | 0.06 | 1 | 1531.0 | 8.42 | .004 | .**005** | 0.01 |
| Time phase | 19.60 | 2 | 1531.0 | 502.47 | < .001 | **< .001** | 0.40 | 24.86 | 2 | 1531.4 | 735.72 | < .001 | **< .001** | 0.49 | 13.92 | 2 | 1531.0 | 1051.80 | < .001 | **< .001** | 0.58 |
| Cluster | 1.95 | 12 | 1551.2 | 8.33 | < .001 | **< .001** | 0.06 | 0.69 | 12 | 1548.0 | 3.42 | < .001 | **< .001** | 0.03 | 0.78 | 12 | 1546.7 | 9.87 | < .001 | **< .001** | 0.07 |
| Maze x Time phase | 5.12 | 2 | 1531.0 | 131.31 | < .001 | **< .001** | 0.15 | 1.03 | 2 | 1531.4 | 30.55 | < .001 | **< .001** | 0.04 | 0.27 | 2 | 1531.0 | 20.63 | < .001 | **< .001** | 0.03 |
| Maze x Cluster | 0.10 | 12 | 1531.0 | 0.42 | .957 | .976 | < 0.01 | 0.23 | 12 | 1531.4 | 1.15 | .317 | .366 | 0.01 | 0.09 | 12 | 1531.0 | 1.09 | .362 | .415 | 0.01 |
| Time phase x Cluster | 5.71 | 24 | 1531.0 | 12.19 | < .001 | **< .001** | 0.16 | 6.29 | 24 | 1531.4 | 15.50 | < .001 | **< .001** | 0.20 | 4.67 | 24 | 1531.0 | 29.42 | < .001 | **< .001** | 0.32 |
| Maze x Time phase x Cluster | 1.20 | 24 | 1531.0 | 2.56 | < .001 | **< .001** | 0.04 | 0.42 | 24 | 1531.4 | 1.04 | .413 | .461 | 0.02 | 0.35 | 24 | 1531.0 | 2.19 | < .001 | .**001** | 0.03 |
| **maze A vs B** |  |  |  |  |  |  |  |  |  |  |  |  |  |  |  |  |  |  |  |  |  |
| Maze | 0.09 | 1 | 1532.5 | 4.69 | .030 | **.039** | < 0.01 | 0.25 | 1 | 1530.9 | 14.26 | < .001 | **< .001** | 0.01 | 0.22 | 1 | 1530.7 | 28.34 | < .001 | **< .001** | 0.02 |
| Time phase | 12.61 | 2 | 1532.5 | 335.93 | < .001 | **< .001** | 0.30 | 17.12 | 2 | 1530.9 | 484.35 | < .001 | **< .001** | 0.39 | 16.85 | 2 | 1530.7 | 1085.18 | < .001 | **< .001** | 0.59 |
| Cluster | 2.75 | 12 | 1554.2 | 12.21 | < .001 | **< .001** | 0.09 | 0.52 | 12 | 1550.5 | 2.44 | .004 | **.006** | 0.02 | 0.69 | 12 | 1546.3 | 7.36 | < .001 | **< .001** | 0.05 |
| Maze x Time phase | 4.75 | 2 | 1532.5 | 126.43 | < .001 | **< .001** | 0.14 | 0.73 | 2 | 1530.9 | 20.76 | < .001 | **< .001** | 0.03 | 0.04 | 2 | 1530.7 | 2.25 | .105 | .131 | < 0.01 |
| Maze x Cluster | 0.14 | 12 | 1532.5 | 0.63 | .815 | .856 | < 0.01 | 0.18 | 12 | 1530.9 | 0.82 | .627 | .689 | 0.01 | 0.09 | 12 | 1530.7 | 0.99 | .457 | .508 | 0.01 |
| Time phase x Cluster | 1.78 | 24 | 1532.5 | 3.95 | < .001 | **< .001** | 0.06 | 3.50 | 24 | 1530.9 | 8.25 | < .001 | **< .001** | 0.11 | 3.87 | 24 | 1530.7 | 20.74 | < .001 | **< .001** | 0.25 |
| Maze x Time phase x Cluster | 3.00 | 24 | 1532.5 | 6.66 | < .001 | **< .001** | 0.09 | 1.28 | 24 | 1530.9 | 3.02 | < .001 | **< .001** | 0.05 | 0.15 | 24 | 1530.7 | 0.79 | .755 | .809 | 0.01 |
| **maze R vs C** |  |  |  |  |  |  |  |  |  |  |  |  |  |  |  |  |  |  |  |  |  |
| Maze | 0.15 | 1 | 1531.8 | 9.12 | .003 | **.004** | 0.01 | 0.30 | 1 | 1531.1 | 20.80 | < .001 | **< .001** | 0.01 | 0.06 | 1 | 1531.0 | 11.01 | < .001 | **.001** | 0.01 |
| Time phase | 14.60 | 2 | 1531.8 | 441.21 | < .001 | **< .001** | 0.40 | 13.67 | 2 | 1531.1 | 470.07 | < .001 | **< .001** | 0.38 | 13.55 | 2 | 1531.0 | 1174.57 | < .001 | **< .001** | 0.61 |
| Cluster | 2.25 | 12 | 1550.7 | 11.32 | < .001 | **< .001** | 0.09 | 0.29 | 12 | 1544.7 | 1.68 | .064 | .081 | 0.01 | 0.29 | 12 | 1542.3 | 4.21 | < .001 | **< .001** | 0.03 |
| Maze x Time phase | 2.29 | 2 | 1531.8 | 69.31 | < .001 | **< .001** | 0.03 | 0.30 | 2 | 1531.1 | 10.47 | < .001 | **< .001** | 0.01 | < 0.01 | 2 | 1531.0 | 0.03 | .972 | .986 | < 0.01 |
| Maze x Cluster | 0.64 | 12 | 1531.8 | 3.25 | < .001 | **< .001** | 0.01 | 0.75 | 12 | 1531.1 | 4.32 | < .001 | **< .001** | 0.03 | 0.05 | 12 | 1531.0 | 0.79 | .664 | .726 | 0.01 |
| Time phase x Cluster | 4.47 | 24 | 1531.8 | 11.26 | < .001 | **< .001** | 0.14 | 5.28 | 24 | 1531.1 | 15.13 | < .001 | **< .001** | 0.19 | 3.72 | 24 | 1531.0 | 26.88 | \| < .001 \| \| --- \| \| < .001 \| | **< .001** | 0.30 |
| Maze x Time phase x Cluster | 1.30 | 24 | 1531.8 | 3.29 | < .001 | **< .001** | 0.07 | 1.30 | 24 | 1531.1 | 3.73 | < .001 | **< .001** | 0.06 | 0.09 | 24 | 1531.0 | 0.64 | .912 | .934 | 0.01 |
| **Pairs relying not** **on the same reference frame** | | | | | | | | | | | | | | | | | | | | | |
| **maze R vs S** |  |  |  |  |  |  |  |  |  |  |  |  |  |  |  |  |  |  |  |  |  |
| Maze | 0.01 | 1 | 1531.8 | 0.72 | .398 | .447 | < 0.01 | 0.09 | 1 | 1531.5 | 5.59 | .018 | **.024** | < 0.01 | 0.15 | 1 | 1531.3 | 22.11 | < .001 | **< .001** | 0.01 |
| Time phase | 18.00 | 2 | 1531.8 | 476.64 | < .001 | **< .001** | 0.38 | 21.88 | 2 | 1531.5 | 686.61 | < .001 | **< .001** | 0.47 | 12.46 | 2 | 1531.3 | 947.02 | < .001 | **< .001** | 0.55 |
| Cluster | 1.43 | 12 | 1551.8 | 6.32 | < .001 | **< .001** | 0.05 | 0.81 | 12 | 1546 | 4.22 | < .001 | **< .001** | 0.03 | 0.53 | 12 | 1546.7 | 6.75 | < .001 | **< .001** | 0.05 |
| Maze x Time phase | 4.97 | 2 | 1531.8 | 131.66 | < .001 | **< .001** | 0.15 | 1.72 | 2 | 1531.5 | 53.85 | < .001 | **< .001** | 0.07 | 0.26 | 2 | 1531.3 | 20.13 | < .001 | **< .001** | 0.03 |
| Maze x Cluster | 0.14 | 12 | 1531.8 | 0.63 | .816 | .856 | < 0.01 | 0.27 | 12 | 1531.5 | 1.41 | .152 | .184 | 0.01 | 0.19 | 12 | 1531.3 | 2.39 | .005 | **.007** | 0.02 |
| Time phase x Cluster | 5.25 | 24 | 1531.8 | 11.58 | < .001 | **< .001** | 0.15 | 6.27 | 24 | 1531.5 | 16.40 | < .001 | **< .001** | 0.20 | 4.71 | 24 | 1531.3 | 29.83 | < .001 | **< .001** | 0.32 |
| Maze x Time phase x Cluster | 1.02 | 24 | 1531.8 | 2.26 | < .001 | **.001** | 0.03 | 0.46 | 24 | 1531.5 | 1.20 | .231 | .269 | 0.02 | 0.35 | 24 | 1531.3 | 2.19 | <.001 | **.001** | 0.03 |
| **maze R vs A** |  |  |  |  |  |  |  |  |  |  |  |  |  |  |  |  |  |  |  |  |  |
| Maze | 0.13 | 1 | 1533.2 | 7.38 | .007 | **.009** | < 0.01 | 0.13 | 1 | 1531.1 | 7.75 | .005 | **.008** | 0.01 | 0.10 | 1 | 1531 | 13.61 | < .001 | **< .001** | 0.01 |
| Time phase | 11.32 | 2 | 1533.2 | 311.50 | < .001 | **< .001** | 0.29 | 14.64 | 2 | 1531.1 | 437.97 | < .001 | **< .001** | 0.36 | 15.36 | 2 | 1531 | 996.92 | < .001 | **< .001** | 0.57 |
| Cluster | 2.04 | 12 | 1554.8 | 9.37 | < .001 | **< .001** | 0.07 | 0.54 | 12 | 1547.9 | 2.70 | .001 | **.002** | 0.02 | 0.42 | 12 | 1546.1 | 4.51 | < .001 | **< .001** | 0.03 |
| Maze x Time phase | 4.29 | 2 | 1533.2 | 118.10 | < .001 | **< .001** | 0.13 | 0.92 | 2 | 1531.1 | 27.66 | < .001 | **< .001** | 0.03 | 0.06 | 2 | 1531 | 3.97 | .019 | **.025** | 0.01 |
| Maze x Cluster | 0.40 | 12 | 1533.2 | 1.85 | .036 | **.046** | 0.01 | 0.27 | 12 | 1531.1 | 1.36 | .179 | .216 | 0.01 | 0.19 | 12 | 1531 | 2.05 | .018 | **.023** | 0.02 |
| Time phase x Cluster | 1.68 | 24 | 1533.2 | 3.85 | < .001 | **< .001** | 0.06 | 3.48 | 24 | 1531.1 | 8.68 | < .001 | **< .001** | 0.12 | 3.90 | 24 | 1531 | 21.08 | < .001 | **< .001** | 0.25 |
| Maze x Time phase x Cluster | 2.47 | 24 | 1533.2 | 5.66 | < .001 | **< .001** | 0.08 | 1.32 | 24 | 1531.1 | 3.29 | < .001 | **< .001** | 0.05 | 0.15 | 24 | 1531 | 0.81 | .722 | .777 | 0.01 |
| **maze R vs B** |  |  |  |  |  |  |  |  |  |  |  |  |  |  |  |  |  |  |  |  |  |
| Maze | < 0.01 | 1 | 1532.1 | 0.26 | .611 | .675 | < 0.01 | 0.02 | 1 | 1531.7 | 1.35 | .246 | .285 | < 0.01 | 0.02 | 1 | 1531.2 | 3.35 | .067 | .085 | < 0.01 |
| Time phase | 23.24 | 2 | 1532.1 | 626.81 | < .001 | **< .001** | 0.45 | 15.45 | 2 | 1531.7 | 514.97 | < .001 | **< .001** | 0.40 | 14.97 | 2 | 1531.2 | 1187.79 | < .001 | **< .001** | 0.61 |
| Cluster | 1.78 | 12 | 1552.2 | 8.00 | < .001 | **< .001** | 0.06 | 0.55 | 12 | 1548.2 | 3.08 | < .001 | **< .001** | 0.02 | 0.38 | 12 | 1544.7 | 5.02 | < .001 | **< .001** | 0.04 |
| Maze x Time phase | 0.04 | 2 | 1532.1 | 0.96 | .383 | .435 | < 0.01 | 0.10 | 2 | 1531.7 | 3.33 | .036 | **.046** | < 0.01 | 0.05 | 2 | 1531.2 | 3.76 | .023 | **.030** | < 0.01 |
| Maze x Cluster | 0.17 | 12 | 1532.1 | 0.76 | .697 | .755 | 0.01 | 0.09 | 12 | 1531.7 | 0.51 | .909 | .934 | < 0.01 | 0.05 | 12 | 1531.2 | 0.67 | .786 | .838 | 0.01 |
| Time phase x Cluster | 6.26 | 24 | 1532.1 | 14.07 | < .001 | **< .001** | 0.18 | 5.85 | 24 | 1531.7 | 16.26 | < .001 | **< .001** | 0.20 | 4.24 | 24 | 1531.2 | 28.03 | < .001 | **< .001** | 0.31 |
| Maze x Time phase x Cluster | 0.18 | 24 | 1532.1 | 0.41 | .995 | .999 | 0.01 | 0.14 | 24 | 1531.7 | 0.40 | .996 | .999 | 0.01 | 0.05 | 24 | 1531.2 | 0.35 | .999 | .999 | 0.01 |
| **maze C vs S** |  |  |  |  |  |  |  |  |  |  |  |  |  |  |  |  |  |  |  |  |  |
| Maze | 0.25 | 1 | 1531.0 | 14.84 | < .001 | **< .001** | 0.01 | 0.72 | 1 | 1530.8 | 44.81 | < .001 | **< .001** | 0.03 | 0.40 | 1 | 1530.8 | 66.92 | < .001 | **< .001** | 0.04 |
| Time phase | 17.53 | 2 | 1531.0 | 510.59 | < .001 | **< .001** | 0.40 | 23.32 | 2 | 1530.8 | 725.68 | < .001 | **< .001** | 0.49 | 12.44 | 2 | 1530.8 | 1038.11 | < .001 | **< .001** | 0.58 |
| Cluster | 2.62 | 12 | 1547.3 | 12.71 | < .001 | **< .001** | 0.09 | 0.42 | 12 | 1542.9 | 2.19 | .010 | **.014** | 0.02 | 0.68 | 12 | 1542.8 | 9.49 | < .001 | **< .001** | 0.07 |
| Maze x Time phase | 0.80 | 2 | 1531.0 | 23.26 | < .001 | **< .001** | 0.03 | 1.00 | 2 | 1530.8 | 31.03 | < .001 | **< .001** | 0.04 | 0.28 | 2 | 1530.8 | 23.59 | < .001 | **< .001** | 0.03 |
| Maze x Cluster | 0.34 | 12 | 1531.0 | 1.66 | .069 | .086 | 0.01 | 0.91 | 12 | 1530.8 | 4.71 | < .001 | **< .001** | 0.04 | 0.10 | 12 | 1530.8 | 1.33 | .196 | .232 | 0.01 |
| Time phase x Cluster | 4.34 | 24 | 1531.0 | 10.53 | < .001 | **< .001** | 0.14 | 5.65 | 24 | 1530.8 | 14.64 | < .001 | **< .001** | 0.19 | 4.15 | 24 | 1530.8 | 28.87 | < .001 | **< .001** | 0.31 |
| Maze x Time phase x Cluster | 1.90 | 24 | 1531.0 | 4.60 | < .001 | **< .001** | 0.07 | 1.64 | 24 | 1530.8 | 4.26 | < .001 | **< .001** | 0.06 | 0.38 | 24 | 1530.8 | 2.66 | < .001 | **< .001** | 0.04 |
| **maze C vs A** |  |  |  |  |  |  |  |  |  |  |  |  |  |  |  |  |  |  |  |  |  |
| Maze | < 0.01 | 1 | 1531.5 | 0.03 | .861 | .895 | < 0.01 | 0.04 | 1 | 1530.5 | 2.15 | .143 | .175 | < 0.01 | 0.01 | 1 | 1530.6 | 0.73 | .394 | .445 | < 0.01 |
| Time phase | 10.60 | 2 | 1531.5 | 323.42 | < .001 | **< .001** | 0.30 | 16.11 | 2 | 1530.5 | 478.31 | < .001 | **< .001** | 0.38 | 15.36 | 2 | 1530.6 | 1085.47 | < .001 | **< .001** | 0.59 |
| Cluster | 3.65 | 12 | 1549.4 | 18.56 | < .001 | **< .001** | 0.13 | 0.79 | 12 | 1544.7 | 3.89 | < .001 | **< .001** | 0.03 | 0.57 | 12 | 1542.4 | 6.74 | < .001 | **< .001** | 0.05 |
| Maze x Time phase | 0.37 | 2 | 1531.5 | 11.31 | < .001 | **< .001** | 0.01 | 0.17 | 2 | 1530.5 | 5.01 | .007 | **.009** | 0.01 | 0.06 | 2 | 1530.6 | 4.15 | .016 | **.021** | 0.01 |
| Maze x Cluster | 0.15 | 12 | 1531.5 | 0.77 | .682 | .742 | 0.01 | 0.30 | 12 | 1530.5 | 1.47 | .127 | .157 | 0.01 | 0.12 | 12 | 1530.6 | 1.44 | .140 | .172 | 0.01 |
| Time phase x Cluster | 2.39 | 24 | 1531.5 | 6.09 | < .001 | **< .001** | 0.09 | 3.61 | 24 | 1530.5 | 8.93 | < .001 | **< .001** | 0.12 | 3.41 | 24 | 1530.6 | 20.06 | < .001 | **< .001** | 0.24 |
| Maze x Time phase x Cluster | 1.71 | 24 | 1531.5 | 4.36 | < .001 | **< .001** | 0.06 | 1.75 | 24 | 1530.5 | 4.34 | < .001 | **< .001** | 0.06 | 0.12 | 24 | 1530.6 | 0.72 | .839 | .876 | 0.01 |
| **maze C vs B** |  |  |  |  |  |  |  |  |  |  |  |  |  |  |  |  |  |  |  |  |  |
| Maze | 0.10 | 1 | 1531.4 | 5.97 | .015 | **.020** | < 0.01 | 0.48 | 1 | 1531.1 | 31.40 | < .001 | **< .001** | 0.02 | 0.16 | 1 | 1530.9 | 26.95 | < .001 | **< .001** | 0.02 |
| Time phase | 16.08 | 2 | 1531.4 | 471.17 | < .001 | **< .001** | 0.38 | 16.01 | 2 | 1531.1 | 524.93 | < .001 | **< .001** | 0.41 | 14.97 | 2 | 1530.9 | 1277.27 | < .001 | **< .001** | 0.63 |
| Cluster | 3.07 | 12 | 1549.6 | 15.00 | < .001 | **< .001** | 0.10 | 0.39 | 12 | 1545.6 | 2.15 | .012 | **.016** | 0.02 | 0.47 | 12 | 1542.8 | 6.73 | < .001 | **< .001** | 0.05 |
| Maze x Time phase | 2.57 | 2 | 1531.4 | 75.21 | < .001 | **< .001** | 0.09 | 0.25 | 2 | 1531.1 | 8.19 | < .001 | **< .001** | 0.01 | 0.05 | 2 | 1530.9 | 4.41 | .012 | **.017** | 0.01 |
| Maze x Cluster | 0.27 | 12 | 1531.4 | 1.30 | .214 | .253 | 0.01 | 0.52 | 12 | 1531.1 | 2.83 | .001 | **.001** | 0.02 | 0.04 | 12 | 1530.9 | 0.56 | .874 | .905 | < 0.01 |
| Time phase x Cluster | 5.06 | 24 | 1531.4 | 12.35 | < .001 | **< .001** | 0.16 | 5.15 | 24 | 1531.1 | 14.07 | < .001 | **< .001** | 0.18 | 3.67 | 24 | 1530.9 | 26.09 | < .001 | **< .001** | 0.29 |
| Maze x Time phase x Cluster | 1.35 | 24 | 1531.4 | 3.30 | < .001 | **< .001** | 0.05 | 1.40 | 24 | 1531.1 | 3.83 | < .001 | **< .001** | 0.06 | 0.11 | 24 | 1530.9 | 0.75 | .803 | .851 | 0.01 |

*Note.* *p.adj denotes p-values adjusted for multiple comparisons using the Benjamini–Hochberg method.*

**Supplementary Table 3**

*Spearman correlation outcome between EEG power and accuracy*

| **Time Point** | **Cluster** |  | **Theta** |  |  | **Alpha** |  |  | **Beta** |  |
| --- | --- | --- | --- | --- | --- | --- | --- | --- | --- | --- |
|  |  | **rho** | ***p*** | ***p.adj*** | **rho** | ***p*** | ***p.adj*** | **rho** | ***p*** | ***p.adj*** |
| Approach | 3 | -0.16 | .076 | .171 | -0.28 | .002 | **.008** | -0.20 | .030 | .081 |
|  | 7 | 0.03 | .746 | .870 | 0.11 | .282 | .454 | -0.14 | .192 | .352 |
|  | 10 | -0.47 | < .001 | **< .001** | -0.13 | .147 | .306 | -0.01 | .948 | .970 |
|  | 12 | -0.57 | < .001 | **< .001** | -0.40 | < .001 | **< .001** | -0.05 | .645 | .785 |
|  | 13 | -0.41 | < .001 | **< .001** | -0.39 | < .001 | **< .001** | 0.06 | .548 | .684 |
| Early Decision | 3 | 0.34 | < .001 | **.001** | 0.03 | .754 | .870 | -0.08 | .409 | .615 |
|  | 7 | 0.33 | .001 | **.005** | 0.02 | .865 | .927 | -0.07 | .490 | .669 |
|  | 10 | 0.32 | < .001 | **.002** | -0.11 | .203 | .352 | -0.10 | .247 | .412 |
|  | 12 | 0.31 | .001 | **.005** | 0.06 | .533 | .684 | 0.01 | .895 | .937 |
|  | 13 | -0.02 | .799 | .899 | -0.14 | .150 | .306 | -0.17 | .075 | .171 |
| Feedback | 3 | -0.26 | .004 | **.012** | -0.12 | .200 | .352 | -0.07 | .431 | .626 |
|  | 7 | -0.60 | < .001 | **< .001** | -0.27 | .007 | **.022** | -0.19 | .059 | .147 |
|  | 10 | -0.26 | .004 | **.012** | -0.02 | .844 | .926 | 0.00 | .973 | .973 |
|  | 12 | -0.34 | < .001 | **.002** | -0.13 | .202 | .352 | 0.06 | .523 | .684 |
|  | 13 | -0.20 | .030 | .081 | 0.08 | .410 | .615 | 0.06 | .491 | .669 |

*Note.* Sample sizes (n) for each cluster were as follows: Cluster 3 (n = 120), Cluster 7 (n = 95), Cluster 10 (n = 125), Cluster 12 (n = 105), and Cluster 13 (n = 115). *p.adj* denotes *p*-values adjusted for multiple comparisons using the Benjamini–Hochberg method.

**Supplementary Table 4**

*Spearman correlation outcome between EEG power and reaction time*

| **Time Point** | **Cluster** |  | **Theta** |  |  | **Alpha** |  |  | **Beta** |  |
| --- | --- | --- | --- | --- | --- | --- | --- | --- | --- | --- |
|  |  | **rho** | ***p*** | ***p.adj*** | **rho** | ***p*** | ***p.adj*** | **rho** | ***p*** | ***p.adj*** |
| Approach | 3 | 0.21 | .023 | **.047** | 0.48 | < .001 | **< .001** | 0.44 | < .001 | **< .001** |
|  | 7 | 0.03 | .746 | .806 | 0.10 | .319 | .411 | 0.39 | < .001 | **.001** |
|  | 10 | 0.31 | < .001 | **< .001** | 0.27 | .002 | **.007** | 0.14 | .131 | .211 |
|  | 12 | 0.49 | < .001 | **< .001** | 0.43 | < .001 | **< .001** | 0.30 | .002 | **.006** |
|  | 13 | 0.27 | .003 | **< .001** | 0.45 | < .001 | **< .001** | 0.09 | .353 | .420 |
| Early Decision | 3 | -0.10 | .254 | .346 | 0.05 | .588 | .678 | 0.16 | .085 | .153 |
|  | 7 | -0.28 | .006 | **.014** | 0.03 | .752 | .806 | 0.24 | .018 | **.038** |
|  | 10 | -0.19 | .038 | .072 | -0.02 | .803 | .821 | 0.00 | .986 | .986 |
|  | 12 | -0.10 | .301 | .399 | 0.04 | .675 | .759 | 0.09 | .355 | .420 |
|  | 13 | 0.30 | .001 | **.005** | 0.27 | .003 | **.009** | 0.13 | .169 | .253 |
| Feedback | 3 | 0.20 | .027 | .053 | 0.13 | .162 | .251 | 0.31 | .001 | **.002** |
|  | 7 | 0.10 | .345 | .420 | 0.16 | .120 | .199 | 0.54 | < .001 | **< .001** |
|  | 10 | 0.26 | .003 | **.009** | 0.11 | .222 | .322 | 0.45 | < .001 | **< .001** |
|  | 12 | 0.16 | .096 | .166 | 0.28 | .004 | **.010** | 0.33 | .001 | **.003** |
|  | 13 | 0.11 | .242 | .340 | 0.03 | .784 | .821 | 0.23 | .012 | **.027** |

*Note.* Sample sizes (n) for each cluster were as follows: Cluster 3 (n = 120), Cluster 7 (n = 95), Cluster 10 (n = 125), Cluster 12 (n = 105), and Cluster 13 (n = 115). *p.adj* denotes *p*-values adjusted for multiple comparisons using the Benjamini–Hochberg method.

**Supplementary Figure 4**

*Correlation between EEG power and accuracy*


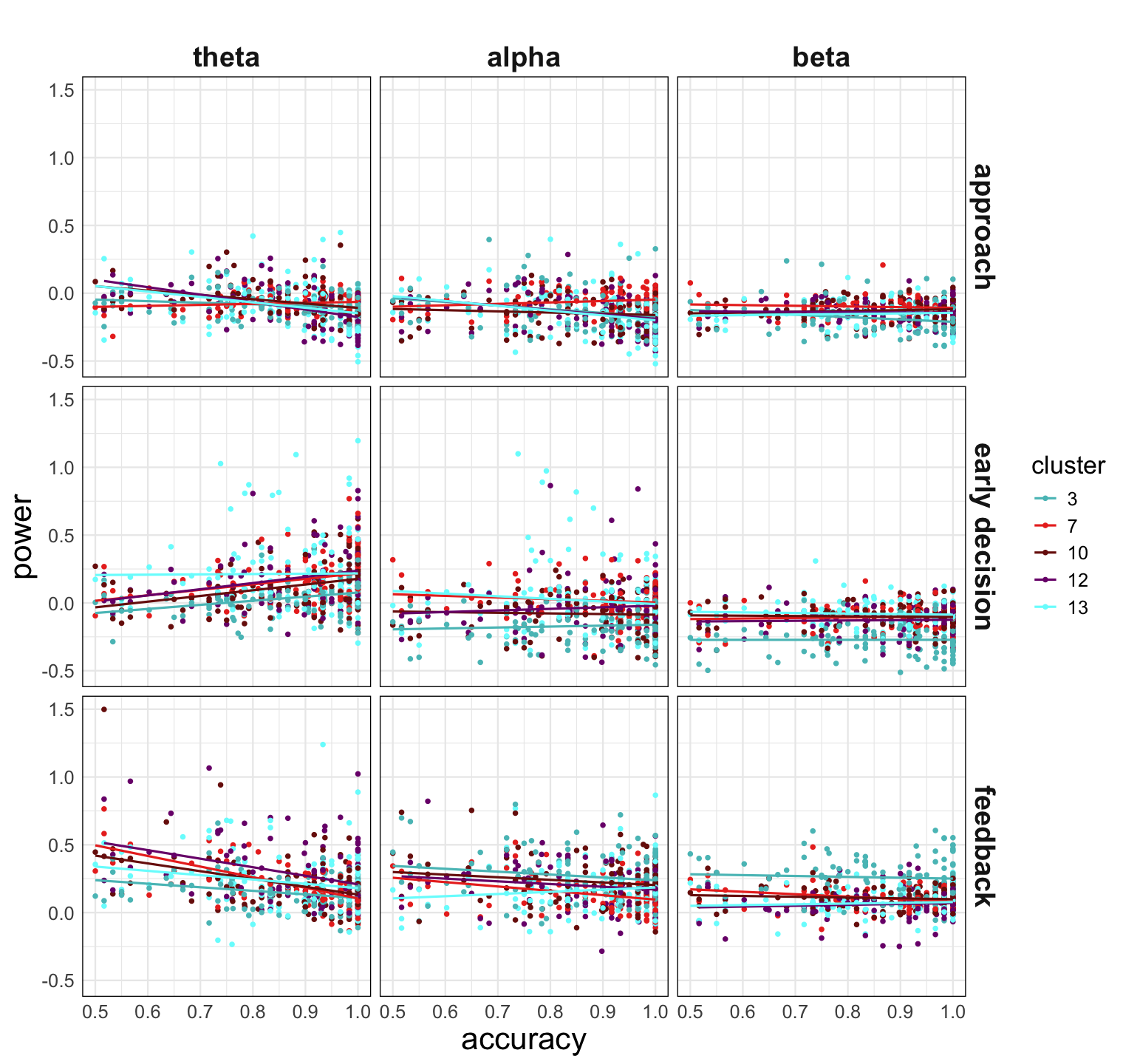


*Note.* Each dot represents the correlation between EEG power and a subject’s average maze accuracy within a given cluster, separated by frequency band and time phase. Cluster 3 corresponds to the frontal region, Cluster 7 to the limbic region, Cluster 10 to the parietal region, Cluster 12 to the temporal region, and Cluster 13 to the occipital region.

**Supplementary Figure 5**

*Correlation between EEG power and reaction time*

**
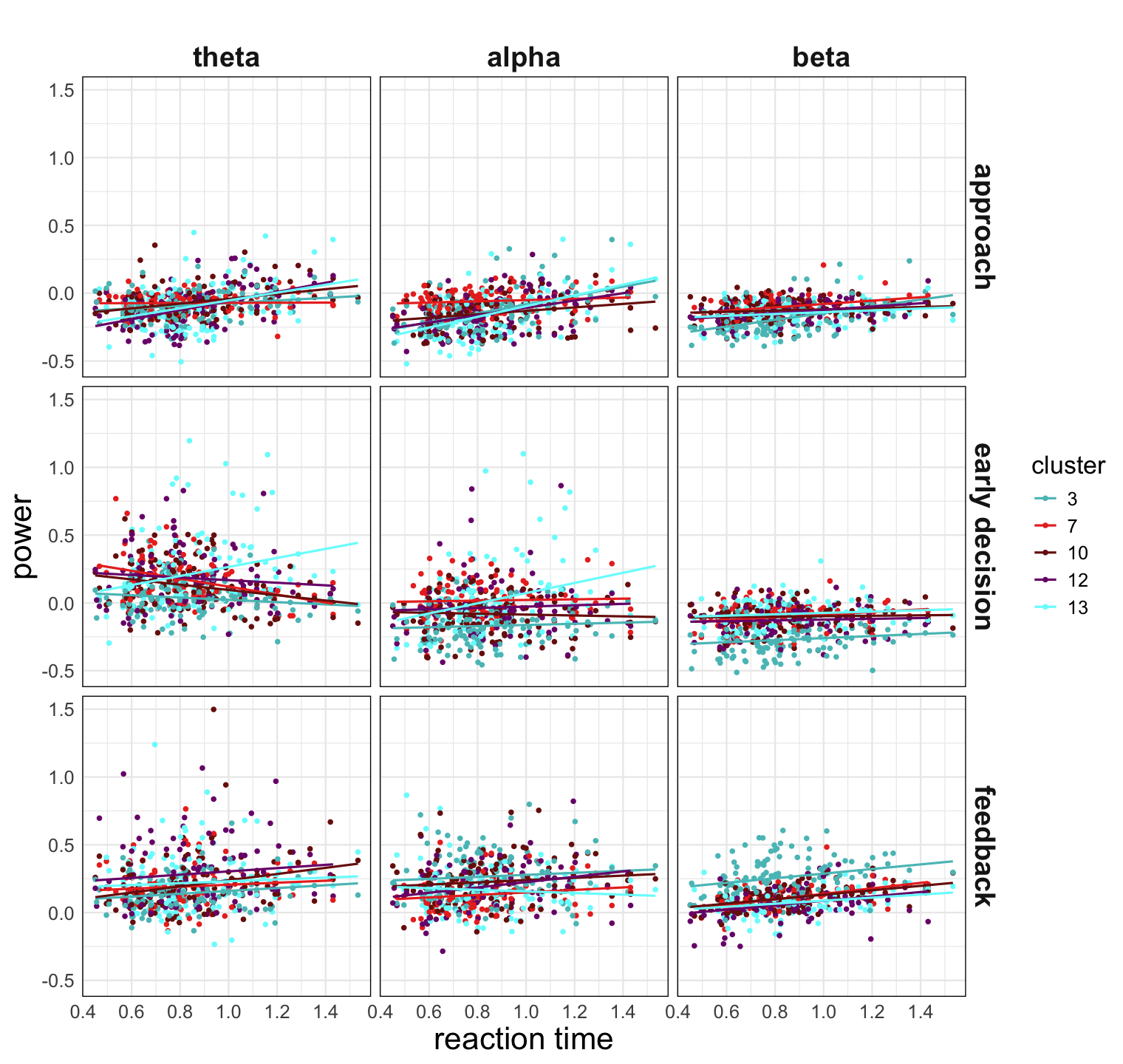
**

*Note.* Each dot represents the correlation between EEG power and a subject’s average maze reaction time within a given cluster, separated by frequency band and time phase. Cluster 3 corresponds to the frontal region, Cluster 7 to the limbic region, Cluster 10 to the parietal region, Cluster 12 to the temporal region, and Cluster 13 to the occipital region.
